# Designing polymer–peptide conjugates to target dipeptide repeat aggregates implicated in amyotrophic lateral sclerosis

**DOI:** 10.1039/d6tb00210b

**Published:** 2026-06-15

**Authors:** Vincent P. Gray, Zixian Cui, Mackenzie Klepsig, Rachel A. Letteri

**Affiliations:** a Department of Chemical Engineering, University of Virginia Charlottesville Virginia 22903 USA rl2qm@virginia.edu

## Abstract

Toxic dipeptide repeats such as the aggregating glycine–alanine (GA)_*n*_ peptide are implicated in the progression of amyotrophic lateral sclerosis (ALS), a lethal neuromuscular disease with an urgent need for new therapeutics. Here, we report polymer–peptide conjugates that prevent aggregation of (GA)_10_. Optical density measurements and transmission electron microscopy demonstrate that conjugates prevent aggregation when co-incubated with (GA)_10_ and disperse pre-aggregated (GA)_10_. These results represent an important step toward a new generation of therapeutics for ALS and contribute to a growing body of literature demonstrating the potential of polymer–peptide conjugates as therapeutics.

## Abstract 2

Polymer-peptide conjugates hinder aggregation of the toxic dipeptide repeat glycine–alanine (GA)_*n*_ peptide that is implicated in the progression of amyotrophic lateral sclerosis, a lethal neuromuscular disease with an urgent need for new therapeutics.

## Graphical abstract

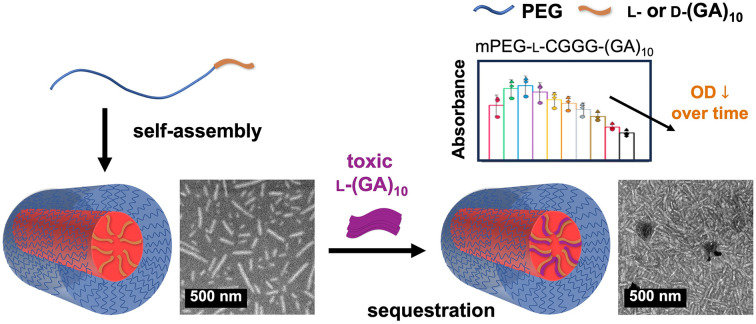

## Introduction

ALS, also known as Lou Gehrig's disease, is a lethal neuromuscular disease.^1–3^ There were approximately 222 000 cases of ALS worldwide in 2015 and that number is estimated to increase by 69% to 376 000 cases by 2040.^4^ Current treatment options include the drugs riluzole^5^ and edaravone,^6^ which slow ALS progression but fail to significantly extend patient survival,^5,7^ highlighting the urgent need for new ALS therapies.

**Scheme 1:**
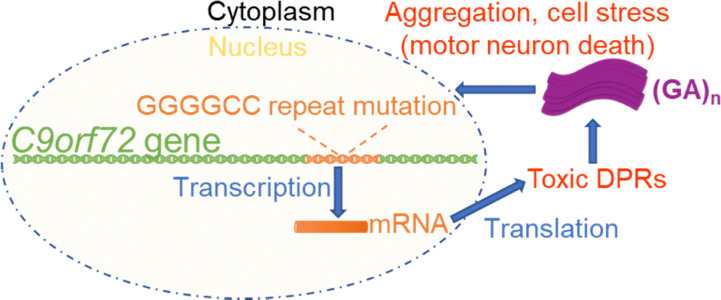
ALS-associated C9orf72 gene mutation. A hexanucleotide expansion of GGGGCC occurs within the gene, which, upon transcription and translation, results in aggregation of repeating hexanucleotide RNA foci, loss of C9orf72 protein, and translation of toxic DPRs, as shown here. Toxic (GA)_*n*_ DPRs aggregate, capture proteins, and cause cellular stress, leading to motor neuron death.

In 40–50% of familial and 5–10% of sporadic cases of ALS,^8^ a mutation of the *C9orf72* gene results in a hexanucleotide expansion of guanine and cytosine. This particular hexanucleotide, GGGGCC, is typically repeated up to 23 times in healthy individuals, but in patients with ALS it is repeated hundreds or thousands of times.^9,10^ This DNA alteration has multiple downstream effects, including: (a) the accumulation of repeating hexanucleotide RNA foci,^10^ (b) the loss of the C9orf72 protein that would normally be encoded by this DNA,^11^ and (c) the production of dipeptide repeat proteins (DPRs), including glycine–alanine (GA)_*n*_, glycine–arginine (GR)_*n*_, proline–arginine (PR)_*n*_, glycine–proline (GP)_*n*_, and proline–alanine (PA)_*n*_ (Scheme 1).^12,13^

While the full pathogenic mechanism of the *C9orf72* mutation is not yet fully understood, and may be a result of all three effects, there is a strong correlation between the (GA)_*n*_ DPR and ALS. The (GA)_*n*_ DPR is present in characteristic inclusions in the affected brain regions of patients with ALS^14^ and is toxic in cell culture.^15,16^ The toxicity of the (GA)_*n*_ DPR may be attributable to the fact that, similar to the amyloid β protein implicated in Alzheimer's disease, (GA)_*n*_ is an aggregating protein.^16^ (GA)_*n*_ DPRs form insoluble cytoplasmic aggregates that transmit between cells^17^ and are toxic to cultured cells and primary neurons, including by toxic mechanisms such as autophagy abnormalities and endoplasmic reticulum (ER) stress.^16,18,19^ Therefore, a method for eliminating aggregating (GA)_*n*_ provides a compelling therapeutic opportunity.

Recent therapeutic approaches focused on designing biologics to sequester or prevent the production of DPRs have had success in mitigating the deleterious hallmarks of ALS.^17,20,21^ For example, antibodies engineered to target (GA)_*n*_ reduced (GA)_*n*_ aggregation both *in vitro* and *in vivo*^17,20^ and, more recently, afforded favorable behavioral outcomes in ALS mouse models.^20^ In addition, mice treated with antibodies had a significant increase in survival compared to untreated mice.^20^ Antisense oligonucleotides targeting the DPR-coding RNA reduced levels of RNA foci and DPRs, which resulted in recovery from cognitive deficits in a mouse model.^21^

While the above examples highlight the transformative therapeutic potential of DPR sequestration for ALS patients, the use of biologics presents several key challenges including the time- and cost-intensive production processes,^22,23^ batch-to-batch variability,^24^ susceptibility to physicochemical alterations during manufacture and transport,^25^ enzymatic degradation,^22,25^ and the need for extensive use of cold chain that would hinder deployment of these treatments (particularly in developing countries).^26^

**Scheme 2:**
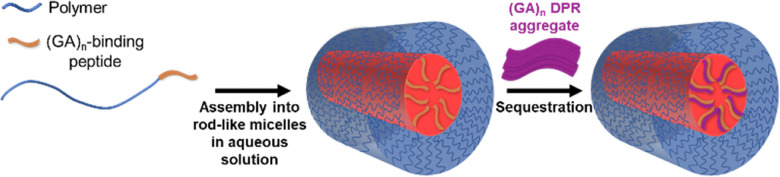
Proposed method to target (GA)_*n*_ DPRs by interaction with a conjugate material composed of a (GA)_*n*_-targeting peptide attached to a polymer.

Inspired by the dispersion of amyloid β aggregates with synthetic peptide–polymer conjugates comprised of short amyloid β-derived peptide and water-soluble comb polymer,^27–29^ here we employ a similar strategy to disperse (GA)_*n*_ DPR aggregates (Scheme 2). Additionally, to leverage documented specific interactions between l-peptides (the stereochemical configuration that is naturally occurring) and their d-peptide enantiomers,^30–32^ we investigate both l- and d- peptides in the conjugates. While amyloid β and other amyloid-forming proteins have been extensively studied, (GA)_*n*_ forms aggregates with properties distinct from model amyloids.^33,34^ Short (GA)_*n*_ DPRs (*n* = 3–6) aggregate as irregular fibrils and sheets,^33^ whereas longer (*n* = 30) DPRs form fibrils, but unlike most amyloids do not bind Thioflavin.^34^ These differences highlight the need for methods to disperse these unique aggregates.

## Results and discussion

### (GA)_10_ recapitulates the features of toxic (GA)_*n*_ and is a suitable model toxic DPR

**Fig. 1:**
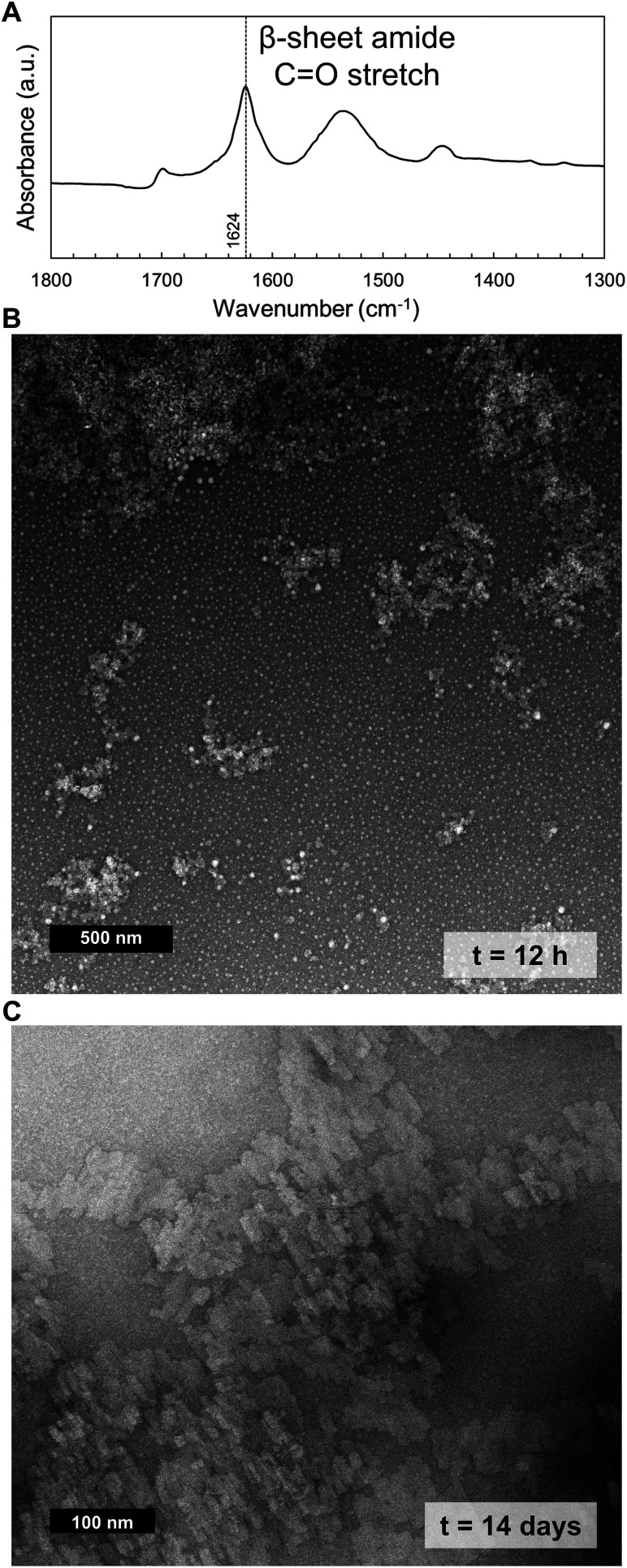
Characterization of the DPR model peptide (GA)_10_. (A) IR spectrum of 3 mg mL^−1^ (GA)_10_ in 10 mM phosphate buffer (pH 7.4) showing a characteristic β-sheet peak at 1624 cm^−1^. TEM image of (GA)_10_ after (B) overnight and (C) 14 day incubation in 10 mM phosphate buffer. While initially amorphous, these aggregates become sheet-like over 14 days. Scale bars are 500 nm and 100 nm, respectively.

We began by synthesizing and characterizing a model (GA)_*n*_ DPR ((GA)_10_) that will allow us to test methods of preventing aggregation and dispersing pre-formed aggregates. We selected *n* = 10 repeats, since while the properties of (GA)_*n*_ are length dependent, neural toxicity and aggregation was seen with *n* = 6,^33^ and *n* = 10 was short enough to be synthesized using solid-phase methods and just soluble enough in aqueous solution (∼0.5 mg mL^−1^, 0.35 mM) to allow for solution characterization. Nuclear magnetic resonance (NMR) spectroscopy, matrix-assisted laser desorption ionization time-of-flight (MALDI-TOF) mass spectrometry, and high-performance liquid chromatography (HPLC) confirmed the primary structure and purity of (GA)_10_ (Fig. S1). To confirm that (GA)_10_ is of sufficient length to recapitulate the secondary structure and aggregation of native (GA)_*n*_ species, we characterized (GA)_10_ using Fourier-transform infrared spectroscopy (FTIR) and transmission electron microscopy (TEM). FTIR of (GA)_10_ in both powder form (Fig. S2) and in 10 mM phosphate buffer (Fig. 1A) shows a strong peak in the amide I band region (1624 cm^−1^) characteristic of β-sheet formation.^35^ As for aggregation, adding (GA)_10_ at 3 mg mL^−1^ (2.1 mM) to 10 mM phosphate buffer results in a visually turbid solution within seconds (Fig. S3). After incubation overnight, TEM shows amorphous aggregates (Fig. 1B and Fig. S4–S6). Yet, after 14 days, (GA)_10_ forms more ordered, sheet-like aggregates like those reported for neurotoxic (GA)_6_ (Fig. 1c and Fig. S16).^33^ That (GA)_10_ captures the β-sheet secondary structure content and aggregation behavior of neurotoxic DPRs suggests that (GA)_10_ is a suitable model for toxic (GA)_*n*_ DPRs.

### Design, synthesis, and characterization of (GA)_*n*_-targeting polymer–peptide conjugates

We designed (GA)_*n*_-targeting polymer–peptide conjugates to maximize interactions between the peptide component and (GA)_*n*_, while maintaining solubility and biocompatibility. Similar to the peptide on the amyloid β-dispersing conjugates,^27–29^ which was taken from the hydrophobic region of the N-terminal domain of amyloid β,^36,37^ we modelled our (GA)_*n*_-binding peptide off (GA)_*n*_ itself, producing conjugates employing either d- or l-(GA)_10_ as the peptide component. As the polymer component of our conjugates, we selected poly(ethylene glycol) (PEG) for its aqueous solubility and well-established safety profile, as evidenced by its use in multiple FDA-approved therapeutics.^38–40^ We anticipated this combination of strongly interacting peptide and soluble polymer to sequester (GA)_*n*_ DPRs without the conjugate becoming too hydrophobic and contributing to aggregation itself.^36^

**Fig. 2:**
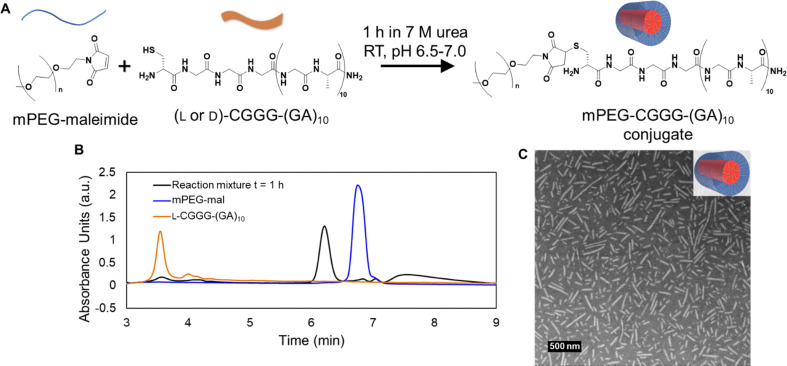
Synthesis and characterization of mPEG-CGGG-(GA)_10_ conjugates. (A) Synthesis of mPEG-CGGG-(GA)_10_ conjugates *via* thiol-maleimide chemistry. (B) HPLC chromatogram showing the reaction mixture after 1 h (black trace), mPEG-mal alone (blue trace), and l-CGGG-(GA)_10_ alone (orange trace). After 1 h of reaction, the largest peak in the reaction mixture (at 6.1 min) is a new peak, attributed to the newly formed conjugate, and elutes between the polymer peak and the peptide peak. The other major peak in the chromatogram, a very broad peak that elutes from 7.2 to 9 min, is attributed to conjugate product that interacts with unreacted peptide. (C) TEM image of mPEG-l-CGGG-(GA)_10_ after overnight incubation in 10 mM phosphate buffer. A rod-like morphology is observed throughout the sample, suggesting as drawn in the inset a cylindrical micelle morphology for the conjugate.

To conjugate (GA)_10_ to PEG, we employed a thiol-maleimide reaction (Fig. 2A). A thiol was added to (GA)_10_ by addition of a cysteine residue at the N-terminus. We also included three glycine residues as a spacer to separate the (GA)_10_ from the polymer and allow it to be more flexible. This resulted in a final sequence of H_2_N-CGGG-(GA)_10_-NH_2_. We note that a 2 : 1 molar ratio of thiol (peptide): maleimide (polymer) was required to push this reaction to completion (see Section S4 of the SI for more details). After the 1 h reaction, HPLC shows a reduction of the peptide peak, the disappearance of the polymer peak, and the appearance of two new peaks (Fig. 2B), one corresponding to the conjugate and one broad peak (from ∼7.2 min to 9 min) that could possibly be newly formed conjugate sequestering unreacted CGGG-(GA)_10_ peptide in solution (see Fig. S7 and the associated discussion). We used preparative-scale HPLC to isolate the conjugate peak and confirmed it to be mPEG-CGGG-(GA)_10_ conjugate by NMR spectroscopy (Fig. S8A). Size exclusion chromatography (SEC) and HPLC (Fig. S8B and C) confirmed conjugate purity. We produced both l- and d-CGGG-(GA)_10_ conjugates in this manner and characterized them by TEM following overnight incubation in 10 mM phosphate buffer (Fig. 2C, Fig. S9 and S10). The presence of a rod-like morphology throughout the sample suggests assembly of the conjugates into cylindrical micelles with a hydrophilic PEG exterior and a hydrophobic (GA)_10_ interior.

### Co-incubation of (GA)_10_ with mPEG-CGGG-(GA)_10_ conjugates leads to aggregate dispersal

**Fig. 3:**
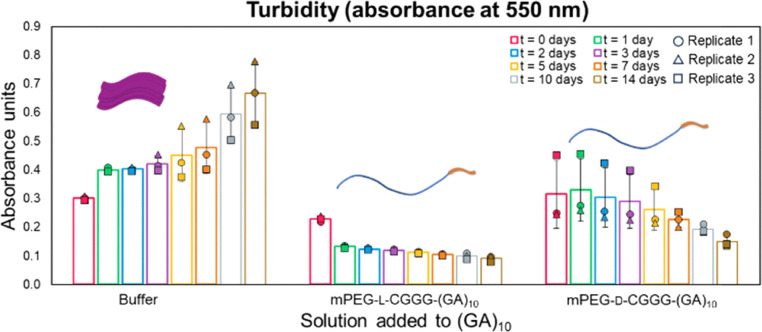
OD over time for (GA)_10_ co-incubated with three different solutions: 10 mM phosphate buffer alone, mPEG-l-CGGG-(GA)_10_, and mPEG-d-CGGG-(GA)_10_ (conjugate concentration = 1 mM). Incubating (GA)_10_ in buffer alone, leads to OD that increases with time. The mPEG-l-CGGG-(GA)_10_ solution led to a decrease in (GA)_10_ OD with a sharp drop over the first day, followed by sustained lowered OD for the remainder of the experiment. Finally, the mPEG-d-CGGG-(GA)_10_ solution initially yielded no change in OD, but after ∼7 days led to a decrease in OD.

We next tested the ability of our conjugates to prevent and reverse (GA)_10_ aggregation by incubating (GA)_10_ with either buffer or a solution containing conjugate. As a measure of aggregation, we used the optical density (OD) of suspensions over time to gauge turbidity by monitoring absorbance at 550 nm (Fig. 3). We settled on OD rather than fluorescence measurements that are also common for assessing amyloid-like peptide assemblies, since (GA)_*n*_ does not always bind thioflavin T^34^ and we found thioflavin T fluorescence served as a probe for total, rather than aggregated, (GA)_10_ concentration (see Section S8 in the SI for more details).

We first sought to ensure that conjugate assembly or microbial growth in buffer did not increase OD. The absorbance of the conjugates alone in buffer is similar to that of buffer alone (Fig. S11), so despite assembly into nanoscale cylinders, the addition of conjugate to the suspension does not increase OD. To determine whether bacterial growth contributes to a rise in OD over the 14 day timespan of the aggregation experiments, we monitored the OD of a solution of 10 mM phosphate buffer for a duration of 28 days and did not observe an increase (Fig. S12).

As expected, adding 10 mM phosphate buffer to (GA)_10_ powder yields continuous increases in OD and heterogeneity between vials over time due to aggregation. Similarly, when we incubate (GA)_10_ with polymer alone, the OD continually increases with time (Fig. S13). However, when adding mPEG-l-CGGG-(GA)_10_ conjugate to (GA)_10_ (molar ratio of conjugate: (GA)_10_ = 0.5) there is a sharp drop in OD over the first day, followed by sustained incremental decreases in OD over the 14 day experiment, demonstrating that the conjugate prevents aggregation.

Co-incubation of (GA)_10_ with the mPEG-d-CGGG-(GA)_10_ conjugate leads to higher average initial (*t* = 0) OD than the mPEG-l-CGGG-(GA)_10_ conjugate. Despite starting with greater OD, the d-conjugate sample does still decrease in OD over time. Yet, while the l-conjugate treated sample dropped OD to ∼50% of its *t* = 0 value after just 1 day, the d-conjugate treated sample required 14 days to gradually drop to a similar ∼50% of its initial OD. This result may indicate different packing of the l- and d-stereochemistries, requiring different time scales for l-(GA)_*n*_ dispersion. While requiring different time scales, these data indicate that both conjugates effectively prevent aggregation.

### Morphological characterization supports that conjugates prevent (GA)_10_ aggregation

**Fig. 4:**
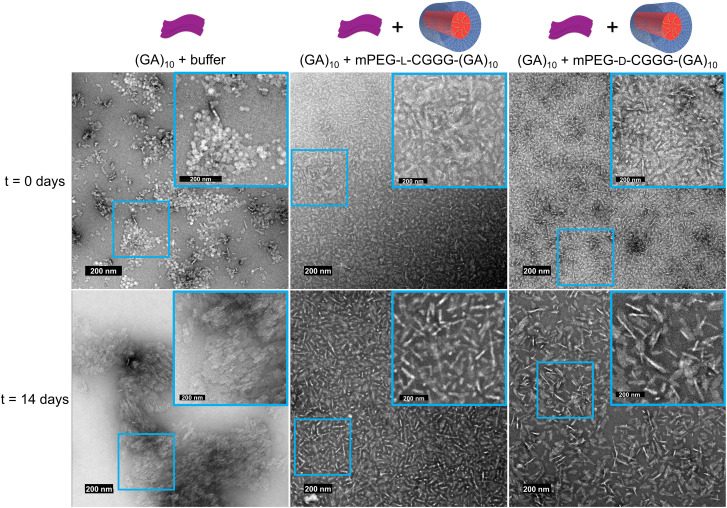
TEM images of (GA)_10_ incubated in buffer alone or co-incubated with 7 mg mL^−1^ mPEG-l-CGGG-(GA)_10_ or 7 mg mL^−1^ mPEG-d-CGGG-(GA)_10_ immediately after incubation began and after 14 days of incubation. In the buffer treatment group, small aggregates of (GA)_10_ are observed coming together to form larger aggregates at the beginning of the experiment, while at the end of the experiment much larger aggregates are observed. For both conjugate conditions, small (GA)_10_ aggregates are observed at the beginning and end of the experiment, but the conjugate treatment prevents them from forming large-scale aggregates as observed for (GA)_10_ incubated in buffer alone.

At the beginning and end of the turbidity experiments, aliquots of each suspension were examined by TEM to investigate changes in the morphological characteristics of the (GA)_10_ (Fig. 4 and Fig. S15–S20). Immediately after adding (GA)_10_ to buffer alone (*t* = 0 days), aggregation occurs, with TEM showing a mixture of small aggregates and larger structures that seem to be made up of groups of those smaller aggregates. To rule out the possibility that the structures were aggregates of the 10 mM phosphate buffer, we took TEM images of the buffer alone as a blank control and, as expected, saw no evidence of any structures formed (Fig. S4). After incubating (GA)_10_ for 14 days in buffer alone, much larger aggregates (on the scale of hundreds to thousands of nm) form, consistent with the increasing OD of the suspension over time. For suspensions of (GA)_10_ treated with conjugate at *t* = 0 days, TEM showed both cylindrical structures consistent with the structures of the conjugates (Fig. 2C, Fig. S10, and S11) and some small aggregates consistent with those in images of (GA)_10_. After 14 days of incubation, we still observe a mixture of both conjugates and small aggregates for both l- and d-conjugates, but there is nothing similar to the large-scale aggregation observed for the (GA)_10_ incubated in buffer alone. The lack of larger aggregates after 14 days is encouraging, and together with the drop in OD over the same timeframe, suggests that the conjugates prevent aggregation of (GA)_10_.

### Dispersal of (GA)_10_ in the presence of conjugates is concentration-dependent

**Fig. 5:**
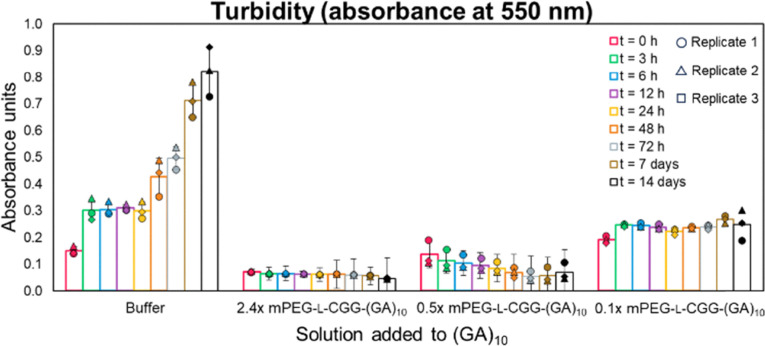
OD over time for (GA)_10_ co-incubated with buffer or three different concentrations of mPEG-l-CGGG-(GA)_10_. As previously observed, the OD of the (GA)_10_ incubated in buffer alone continually increases over time. The (GA)_10_ treated with 35 mg mL^−1^ mPEG-l-CGGG-(GA)_10_ has low OD initially and remains low, indicating that it prevents most aggregation from occurring at all. The 7 mg mL^−1^ mPEG-l-CGGG-(GA)_10_ treatment condition has an initially higher OD that drops over time, indicating that aggregates are not able to grow in the presence of the conjugate. Finally, the (GA)_10_ treated with 0.35 mg mL^−1^ mPEG-l-CGGG-(GA)_10_ starts with a higher OD than the other conditions and has an increase over the first 3 h. The OD in this treatment condition then remains stable for the next 14 days, suggesting that aggregates form and are not broken up, but the conjugate also does not allow them to continue growing as in the buffer condition.

To test the effects of conjugate concentration on (GA)_10_ aggregation prevention and dispersal, we repeated the previous OD experiments, which were conducted with a 0.5 molar ratio of conjugate to (GA)_10_, and treated (GA)_10_ with l-conjugate at higher and lower concentrations, yielding conjugate to molar ratios = 0.1 and 2.4 (Fig. 5). In an effort to capture data during the sharp drop in OD over the first 24 h when treating with mPEG-l-CGGG-(GA)_10_ in the original experiment (Fig. 3), we monitored OD at earlier timepoints (*t* = 3 h, *t* = 6 h, and *t* = 12 h) in addition to some of the same later timepoints. The OD of (GA)_10_ in buffer alone again increases over time and becomes more heterogeneous with time. Treating with 2.4× mPEG-l-CGGG-(GA)_10_ to (GA)_10_ prevented initial aggregation of (GA)_10_ to a significant degree (*p* < 0.005), with the conjugate-treated sample having <50% of the initial OD of (GA)_10_ in buffer and staying consistently low over 14 days. On the other hand, the initial OD of the (GA)_10_ incubated with 0.5× molar equivalents of mPEG-l-CGGG-(GA)_10_ is similar to the initial OD of the (GA)_10_ incubated with buffer alone. However, the OD decreases with time to a final OD similar to that of the 2.4× treatment group. Finally, the OD of (GA)_10_ incubated with the lowest concentration of conjugate (0.1× mPEG-l-CGGG-(GA)_10_ to (GA)_10_) experienced a small increase in OD after 3 h, then remained at the same OD for the duration of the study. These data indicate a concentration-dependent response of (GA)_10_ aggregation to mPEG-l-CGGG-(GA)_10_, where at high concentrations the conjugate prevents initial aggregation and intermediate and low concentrations of the conjugate prevent aggregate growth.

### Incubation of mPEG-CGGG-(GA)_10_ conjugates with pre-aggregated (GA)_10_

**Fig. 6:**
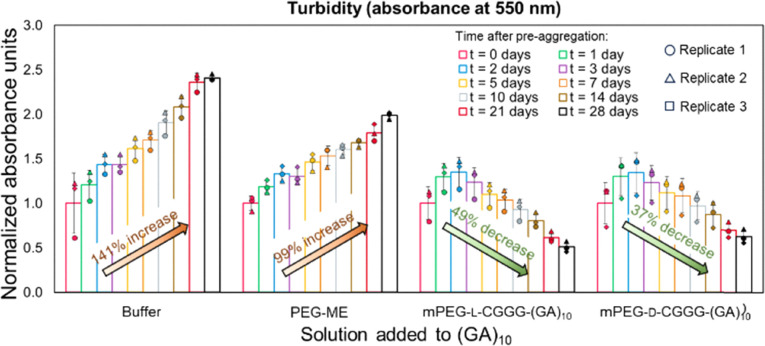
Turbidity over time for pre-aggregated (GA)_10_. (GA)_10_ was allowed to aggregate for 7 days, then treated with either no further additives (‘buffer’), 7 mg mL^−1^ PEG-ME, or 7 mg mL^−1^ mPEG-l-CGGG-(GA)_10_, or 7 mg mL^−1^ mPEG-d-CGGG-(GA)_10_, corresponding to a conjugate: (GA)_10_ molar ratio = 0.5. The time indicates the days since treatment addition, rather than total days of aggregation. The (GA)_10_ incubated with buffer or treated with PEG-ME continually increase in turbidity over time. However, the (GA)_10_ suspensions treated with mPEG-l-CGGG-(GA)_10_ or mPEG-d-CGGG-(GA)_10_ increase in turbidity for the first two days, then decrease in turbidity over 28 days to levels well below the turbidity after pre-aggregation, indicative of the conjugates breaking up the aggregates that formed. Arrows indicate the percent change in turbidity from *t* = 0 days after pre-aggregation to *t* = 28 days after pre-aggregation for each solution.

To better reflect the disease state in ALS, we allowed (GA)_10_ to first aggregate in buffer for 7 days before applying polymer controls (PEG-methyl ether, PEG-ME) or conjugate (mPEG-l-CGGG-(GA)_10_ or mPEG-d-CGGG-(GA)_10_) in powder form. In these experiments, we used a 0.5× conjugate: (GA)_10_ molar ratio. The OD of the solutions was monitored for 28 days after adding PEG-ME or conjugate to observe the continued aggregation behavior of (GA)_10_ (Fig. 6). As expected, the (GA)_10_ incubated in buffer alone or with PEG-ME alone continued to aggregate over time. The two samples treated with conjugate increased in OD over the first 2 days, but thereafter the OD decreased. Whereas initial co-incubation (Fig. 3) showed difference in treating with mPEG-l-CGGG-(GA)_10_ and mPEG-d-CGGG-(GA)_10_, here the two conjugate treatments lead to very similar decreases in (GA)_10_ aggregation. The differences between the two control groups (buffer and PEG-ME alone) and the two treatment groups are clear, with aggregation going unchecked in the control groups whereas aggregation initially accelerates but then slows and reverses for the treatment groups. This is supported by calculating a percent change in OD from *t* = 0 days after pre-aggregation to *t* = 28 days after pre-aggregation. The (GA)_10_ treated with buffer experienced a 141% increase in OD over this time period, the (GA)_10_ treated with PEG-ME experienced a 99% increase in OD, the (GA)_10_ treated with mPEG-l-CGGG-(GA)_10_ experienced a 49% decrease in OD, and the (GA)_10_ treated with mPEG-d-CGGG-(GA)_10_ experienced a 37% decrease in OD. Thus, while our other experiments show that we can prevent aggregation, these experiments show that we can begin to reverse it as well.

**Fig. 7:**
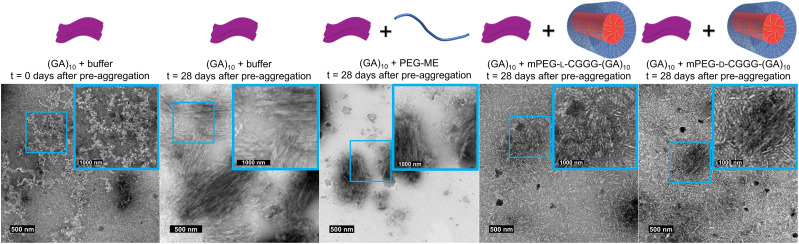
TEM images of (A) (GA)_10_ at the end of the 7-day pre-aggregation period for comparison to (B) pre-aggregated (GA)_10_ incubated in buffer alone, (C) with PEG-ME, (D) with mPEG-l-CGGG-(GA)_10_, and (E) with mPEG-d-CGGG-(GA)_10_ 28 days after the 7 day pre-aggregation period. Matching the trends in the OD data, (GA)_10_ incubated in buffer or PEG-ME forms larger-scale aggregates, while (GA)_10_ incubated in mPEG-l-CGGG-(GA)_10_ or mPEG-d-CGGG-(GA)_10_ show no evidence of similar larger-scale aggregates.

In an analogous manner to the co-incubation OD study, we acquired TEM images at the beginning (*i.e.*, after pre-aggregation and before adding polymer or conjugate) and end of the experiment (28 days) (Fig. 7 and Fig. S21–S25). After the pre-aggregation period, (GA)_10_ formed small aggregates and larger clusters of aggregates (Fig. 7A), then when left untreated for 28 days formed larger-scale aggregates (Fig. 7B). The (GA)_10_ treated with just PEG-ME had a similar morphology to the (GA)_10_ that was incubated in buffer alone (Fig. 7C). Images of the two conjugate-treated suspensions after 28 days (Fig. 7D and E) showed some small aggregates, but no evidence of the larger-scale (GA)_10_ aggregates observed for the (GA)_10_ incubated in PEG-ME or buffer.

## Conclusions

This work introduces a synthetic system designed to target and reduce aggregation of toxic, aggregating (GA)_*n*_ DPRs that are implicated in amyotrophic lateral sclerosis. Polymer–peptide conjugates with a hydrophilic PEG polymer component and a hydrophobic (GA)_10_ peptide component not only stop the aggregation of a model DPR, (GA)_10_, but also disperse pre-formed aggregates. This is supported by decreases in the OD of (GA)_10_ suspensions incubated with d- or l-conjugates over time, whereas the OD of (GA)_10_ solutions incubated with polymer alone or buffer alone increases over time. Additionally, TEM images reveal significant morphological differences, with more extensive aggregation for (GA)_10_ incubated in buffer or with polymer alone compared to (GA)_10_ incubated with conjugates.

The successful dispersion of (GA)_10_ by these polymer–peptide conjugates mirrors the dispersion of Amyloid β by previously reported poly(HPMA)-peptide conjugates.^27,28,37^ Taken together, these data suggest that polymer–peptide conjugates, where the polymer is hydrophilic and biocompatible and the peptide binds to a protein of interest, may be a generalizable platform for targeting and sequestering proteins. In fact, this approach could be employed to target other toxic DPRs such as (GR)_*n*_ and (PR)_*n*_ in future studies, testing the generalizability of the approach beyond amyloid-like proteins.

While we expected that using conjugates with d-peptides might be more effective than conjugates with l-peptides due to previous reports of β-sheet peptides that form stronger interactions between the d and l-enantiomers, we observed a faster drop in OD for the l-conjugates in our co-incubation study, and little difference in the effectiveness of our d- and l-conjugates in preventing and dispersing aggregation in the pre-aggregation study. This suggests that d- and l-(GA)_*n*_ do not interact specifically in the same way that d- and l-peptides studied in previous reports do. These data represent a unique middle ground where the d-peptides in the conjugate clearly interact with the l-(GA)_10_, but do not interact more strongly than l-conjugates. In previous examples with other peptide systems, heterochiral interactions are typically either stronger than homochiral or do not occur at all; the unique interaction between d- and l-(GA)_10_ deserves further investigation to further elucidate the design rules for stereochemistry-directed interactions. Moreover, since both d- and l-conjugates capably disperse aggregates, the d- conjugates can be used if enhanced proteolytic stability is desired since d-peptides resist proteolytic degradation.

Encouragingly, this study furnishes two conjugates capable of dispersing disease state-mimicking aggregates of (GA)_10_. While future directions include investigating the role of polymer chemistry and architecture, peptide length, and efficacy in dispersing aggregates of longer (GA)_*n*_ in biological systems, the design of a synthetic therapeutic that is effective at both preventing and disrupting ALS-associated aggregation is a crucial step toward optimizing ALS therapeutics that are less difficult/expensive to produce and less cold chain-dependent than current biologics.

## Materials and methods

### Materials

Potassium phosphate dibasic (≥98%), potassium phosphate monobasic (≥99.0%), sodium hydroxide (NaOH, 97%) pellets, acetonitrile (HPLC grade), trifluoracetic acid (TFA, 99%), hydrochloric acid (37 wt%), dimethylformamide (DMF, ≥99.8%), diethyl ether (≥99.0%, contains butylated hydroxytoluene as inhibitor), triisopropylsilane (98%), piperidine (≥99%), 2,2′-(ethylenedioxy)diethanethiol (95%), diisopropyl carbodiimde (99%), and methoxypolyethylene glycol maleimide 5000 (≥90%), were purchased from Sigma-Aldrich. Urea (99.0–100.5%) was purchased from Avantor. Rink resin SS (0.51 mmol g^−1^ loading, 100–200 mesh, 1% divinylbenzene), Fmoc-Gly-OH, Fmoc-Ala-OH, Fmoc-d-Ala-OH, Fmoc-Cys(Trt)-OH, Fmoc-d-Cys(Trt)-OH, and Oxyma Pure were purchased from Advanced ChemTech. Water purified by reverse osmosis (RO water) was obtained from an in-house supply and ultrapure water (18.2 MΩ cm) was obtained from a Thermo Scientific Smart2Pure water purification system. All chemicals were used without further purification.

### Peptide synthesis

(GA)_10_, (GA)_20_, l-CGGG-(GA)_10_, and d-CGGG-(GA)_10_ were synthesized on a CEM Corporation Liberty Blue automated, microwave-assisted peptide synthesizer. Synthesis was performed using standard Fmoc methods on a Rink amide resin SS (0.5 mmol g^−1^ substitution, 100–200 mesh, 1% divinylbenzene, Advanced ChemTech). First, the resin was swelled in DMF for 5 min, then two “dummy coupling” steps were performed, designed to add DMF to the reaction vessel and heat to 90 °C like a normal coupling method. These dummy couplings ensure that the instrument is fully warmed up and consistently hitting the target temperature before the actual coupling methods begin. To grow the chain, Fmoc-protected amino acids are added to the reaction vessel and the Fmoc protecting group is removed using 20% (v/v) piperidine in DMF. The coupling reaction is then performed by adding diisopropyl carbodiimide (1 M in DMF) and Oxyma Pure (1 M in DMF) to the reaction vessel and heating to 90 °C for 2 min. The same steps for Fmoc removal and coupling are repeated until the peptide is built from C-terminus to N-terminus.

The peptides were cleaved from the resin and Trt side chain protecting groups were removed from the Cys residues *via* a 3 h, room temperature reaction in a solution of 92.5% TFA, 2.5% triisopropylsilane, 2.5% 2,2′-(ethylenedioxy)diethanethiol, and 2.5% deionized water. Following the reaction, the mixture was precipitated in cold ether and centrifuged (4816 × *g* for 5 min at 4 °C) to obtain a peptide pellet. This pellet was suspended once more in cold ether and centrifuged under the same conditions, before being dried under vacuum for 45 min. Dried peptides were dissolved in RO water, frozen in liquid nitrogen, lyophilized, and stored as powders at −20 °C.

### Nuclear magnetic resonance (NMR) spectroscopy

Peptides or conjugates were dissolved at 5–7 mg mL^−1^ in deuterium oxide (D_2_O) or deuterated dimethyl sulfoxide (DMSO-d6). ^1^H NMR spectroscopy was conducted on a 600 MHz Bruker Avance III spectrometer equipped with a 5 mm HCN Zpfg probe. Chemical shifts were referenced to the solvent residual peak (either 2.50 or 4.79 ppm for DMSO-d6 or D_2_O, respectively).

### Matrix-assisted laser desorption/ionization (MALDI) time-of-flight (TOF) mass spectrometry

MALDI-TOF samples were prepared in RO water at a concentration of ∼1 mg mL^−1^. Samples were mixed 1 : 1 v/v with a 5 mg mL^−1^ solution of cyano-4-hydroxycinnamic acid (CHCA) matrix prepared in 70% (v/v) acetonitrile in water + 0.1% TFA by pipetting up and down 6 times (2 µL of sample + 2 µL of CHCA matrix). A 2 µL aliquot of this solution was pipetted onto a FlexiMass SR48 target plate (Shimadzu) and dried at room temperature. The dried sample was loaded into a Shimadzu 8030 MALDI-TOF instrument, which was calibrated using MALDI TOFMix (LaserBio Labs) calibrant before every use.

### Analytical-scale high performance liquid chromatography (HPLC)

(GA)_10_ was dissolved at 0.5 mg mL^−1^ in HPLC solvent (95% ultrapure water + 0.1% TFA, 5% acetonitrile + 0.1% TFA) and double filtered (0.45 µm polytetrafluoroethylene membranes, 13 mm, VWR) to obtain a clear solution and remove aggregates. d- and l-CGGG-(GA)_10_ were dissolved at 1 mg mL^−1^ in HPLC solvent and double filtered to obtain a clear solution. Samples were loaded into 2 mL vials and HPLC was performed on a Waters Alliance e2695 HPLC system with a 2998 photodiode array detector with separation achieved using an XBridge C18 reverse-phase column (4.6 × 75 mm, 3.5 µm particle size). Peptides were eluted using a 1 mL min^−1^ linear gradient from 5–95% (v/v) acetonitrile + 0.1% TFA in water + 0.1% TFA over 9 min with the column operating at 35 °C. Elution was monitored by absorbance at 214 nm.

### Conjugation of CGGG-(GA)_10_ peptides to poly(ethylene glycol)

A thiol-maleimide reaction was used to conjugate d- or l-CGGG-(GA)_10_ to poly(ethylene glycol) (PEG). Both peptide and polymer were dissolved at a concentration of 1 mM in 7 M urea dissolved in ultrapure water, where urea (a known denaturant)^41^ supports solubilization. Each sample was vortexed for 30 s and sonicated in a sonication bath for 15 s to fully dissolve the peptide or polymer. The pH was then adjusted to 6.5–6.9 for both solutions using 1–5 µL of 1 M NaOH_(aq)_ or 1 M HCl_(aq)_. The pH was kept in this range to favor the thiol-maleimide reaction over an amine-maleimide reaction that becomes more favorable at higher pH. Peptide and polymer solutions were mixed at a volume ratio of 2 : 1 peptide: polymer (6.6 mL of peptide solution to 3.3 mL of polymer solution) to drive the reaction to completion (see Appendix C4.1). The mixture was stirred for 30 s on a Heidolph Hei-plate magnetic stir plate before the pH was checked again to ensure it remained between 6.5–6.9. The reaction mixture was allowed to stir for 1 h at room temperature.

### mPEG-CGGG-(GA)_10_ conjugate purification by preparative-scale HPLC

After 1 h of reaction, the reaction mixture described above was double filtered and directly loaded into the injection loop of a Waters 2545 HPLC system with an attached 2489 photodiode array detector and Waters Fraction Collector III collection system. The mixture was separated on an XBridge C18 reverse-phase column (30 × 150 mm, 5 µm particle size) using a gradient from 5% to 30% acetonitrile in water + 0.1% TFA from 2.22 to 4 min and 30% to 60% acetonitrile in water + 0.1% TFA from 4 min to 18 min. Fractions of eluent were collected in glass culture tubes (13 × 100 mm, VWR) and fractions that eluted from the desired peak were combined and lyophilized. The powders obtained from lyophilization were stored at −20 °C.

### Optical density turbidity experiments: co-incubation

For OD experiments, peptide powder (3.6 mg, 2.8 µmol) was added to 7 mL vials. Separately, solutions of polymer or conjugate were prepared at concentrations of 35, 7, or 0.35 mg mL^−1^ and vortexed to dissolve. To start the experiment, 1.2 mL of buffer, polymer, or conjugate solution was added to the vials containing peptide. Thus, the conjugate solutions prepared at 35, 7, or 0.35 mg mL^−1^ yielded final molar ratios of conjugate: (GA)_10_ DPR molar ratios of approximately 0.02, 0.5, and 2.4, respectively. A stir bar (either 4.3 × 9.4 mm egg-shaped or 3.1 × 12.7 mm cylindrical) was added and solutions were stirred at 300 rpm on a Heidolph Hei-plate magnetic stir plate at room temperature. At the designated timepoints, 3 aliquots of 200 µL were added to a 96 well plate from each vial. Absorbance at 550 nm was measured on a Tecan Infinite M Plex plate reader. After the absorbance measurement, the 200 µL aliquots were returned to stirring in their original vials until the next timepoint. The values reported are the average of three 200 µL aliquots per vial subtracted by the average absorbance of three 200 µL aliquots of 10 mM phosphate buffer.

### Optical density turbidity experiments: pre-aggregation

The OD experiments probing pre-aggregation were conducted in the same way as the co-incubation experiments, with the exception that 1.2 mL of buffer was added to all vials of peptide. From that time, the solutions were stirred under the same conditions for 7 days to allow (GA)_10_ to aggregate in buffer in the absence of polymer or conjugate treatments. After 7 days of incubation in buffer, polymer or conjugate (molar ratio conjugate: (GA)_10_ = 0.5) was added directly to the vials in powder form. The values reported are the average of three 200 µL aliquots per vial subtracted by the average absorbance of three 200 µL aliquots of 10 mM phosphate buffer.

### Thioflavin T fluorescence

For (GA)_10_ thioflavin T fluorescence, we prepared (GA)_10_ solutions at 3, 1.5, 0.75, 0.35, 0.15, and 0.04 mg mL^−1^ in 10 mM phosphate buffer. Each solution was prepared separately rather than by dilution because (GA)_10_ is not fully soluble at all of these concentrations, so a dilution would not be reliable. We then prepared a 10 mM stock solution of thioflavin T and diluted the stock solution into each (GA)_10_ solution to yield a final concentration of 10 µM thioflavin T in each vial. The vials were stirred for 75 min, then fluorescence was measured on a Tecan Infinite M Plex plate reader with an excitation wavelength of 450 nm and an emission wavelength of 482 nm.

For thioflavin T fluorescence measured during the OD study, we prepared a fresh stock solution of thioflavin T at 1 mM for each day a measurement was taken, then diluted this stock to 100 µM. After absorbance at 550 nm was measured for OD, 20 µL of the 100 µM stock solution was added to each 200 µL well in the 96 well plate, for a final concentration of 10 µM thioflavin T in each well. Fluorescence was measured at the same timepoints as the OD experiment on a Tecan Infinite M Plex plate reader with an excitation wavelength of 450 nm and an emission wavelength of 482 nm.

### TEM imaging

To monitor morphological changes during the OD experiment, 10 µL aliquots were taken from one of the vials prepared at each condition at the same timepoints as the OD experiments. Aliquots were stored at −20 °C until sample grids were ready to be prepared. Carbon-coated copper grids (300 mesh, Electron Microscopy Sciences), were pretreated in a plasma cleaner with 20% v/v O_2(g)_ and 80% v/v Ar_(g)_ for 30 s. To apply the samples to the grids, 3 µL of sample were pipetted onto the grids and left for 1 min. To wick away excess solution, filter paper was placed at the edge of the grid, and washed three times by applying and then wicking away 10 µL of RO water. Washed grids were dried for 1 min before adding 3 µL of 2% aqueous uranyl acetate staining solution for 1 min. Excess uranyl acetate solution was wicked away with filter paper, and the samples were air dried. Samples were imaged on an FEI Titan instrument operating at an accelerating voltage of 120 kV at magnifications ranging from 8100× to 34 000×.

## Author contributions

V. G., Z. C., M. K., and R. L. conceived the idea and designed experiments. V. G., Z. C., and M. K. conducted experiments and associated analysis. V. G. and R. L. wrote the manuscript with input from all authors. R. L. acquired funding and supervised the work.

## Conflicts of interest

There are no conflicts to declare.

## Supplementary Material

TB-014-D6TB00210B-s001

## Data Availability

The data supporting this article have been included as part of the supplementary information (SI). Supplementary information: peptide characterization, additional TEM images of (GA)_10_ incubated in buffer and with conjugates, thioflavin T fluorescence of (GA)_10_, OD of conjugates in buffer, OD of buffer over time. See DOI: https://doi.org/10.1039/d6tb00210b.
