# Supplementary material for: Designing polymer–peptide conjugates to target dipeptide repeat aggregates implicated in amyotrophic lateral sclerosis
Source: J Mater Chem B. 2026 Jun 15;14(26):8149–58. doi: 10.1039/d6tb00210b (PMC13267777; doi:10.1039/d6tb00210b)
Supplement: TB-014-D6TB00210B-s001 [file TB-014-D6TB00210B-s001.pdf]

Supplementary Information (SI) for Journal of Materials Chemistry B.
This journal is © The Royal Society of Chemistry 2026

Supporting Information

Designing polymer-peptide conjugates to target dipeptide repeat aggregates implicated in amyotrophic lateral sclerosis

Vincent P. Gray,[1] Zixian Cui,[1] Mackenzie Klepsig,[1] and Rachel A. Letteri[1*]

[1]Department of Chemical Engineering, University of Virginia, Charlottesville, VA 22903

*Corresponding Author: Rachel A. Letteri, e-mail: rl2qm@virginia.edu

Table of Contents

S1. $(GA)_{10}$ characterization 3
- **S1.1 $(GA)_{10}$ structure and purity** 3
- **S1.2 $(GA)_{10}$ secondary structure** 3
- **S1.3 $(GA)_{10}$ forms turbid solutions at 3 mg/mL in 10 mM phosphate buffer** 4

S2. TEM images of 10 mM phosphate buffer 4

S3. Additional TEM images of $(GA)_{10}$ 5

S4. Characterization of mPEG-CGGG-$(GA)_{10}$ conjugates 7
- **S4.1 Molar ratio required for full conversion of thiol-maleimide conjugation reaction** 7
- **S4.2 Structure and purity of mPEG-CGGG-$(GA)_{10}$ conjugates** 8
- **S4.3 Additional TEM images of mPEG-L-CGGG-$(GA)_{10}$** 10
- **S4.4 Additional TEM images of mPEG-D-CGGG-$(GA)_{10}$** 11

S5. Turbidity of mPEG-L-CGGG-$(GA)_{10}$ and mPEG-D-CGGG-$(GA)_{10}$ in 10 mM phosphate buffer 11

S6. Turbidity of 10 mM phosphate buffer over 28 days 12

S7. Selection of a polymer control: Turbidity of $(GA)_{10}$ incubated with mPEG-mal and methyl ether-terminated PEG (PEG-ME) 13

S8. Thioflavin T fluorescence of $(GA)_{10}$ incubated with buffer, mPEG-mal, mPEG-L-CGGG-$(GA)_{10}$, or mPEG-D-CGGG-$(GA)_{10}$ 13

S9. Additional TEM images of coincubation study of $(GA)_{10}$ with buffer, mPEG-L-CGGG-$(GA)_{10}$, and mPEG-D-CGGG-$(GA)_{10}$ 15
- **S9.1 Additional TEM images of $(GA)_{10}$ incubated in buffer at t = 0 days** 15
- **S9.2 Additional TEM images of $(GA)_{10}$ incubated in buffer at t = 14 days** 16
- **S9.3 Additional TEM images of $(GA)_{10}$ incubated with mPEG-L-CGGG-$(GA)_{10}$ at t = 0 days** 17

S9.4 Additional TEM images of $(GA)_{10}$ incubated with mPEG-L-CGGG-$(GA)_{10}$ at t = 14 days 18

S9.5 Additional TEM images of $(GA)_{10}$ incubated with mPEG-D-CGGG-$(GA)_{10}$ at t = 0 days 19

S9.6 Additional TEM images of $(GA)_{10}$ incubated with mPEG-D-CGGG-$(GA)_{10}$ at t = 14 days 20

S10. Additional TEM images of pre-aggregation study of $(GA)_{10}$ with buffer, PEG-ME, mPEG-L-CGGG-$(GA)_{10}$, and mPEG-D-CGGG-$(GA)_{10}$ 21

S10.1 Additional TEM images of $(GA)_{10}$ pre-aggregated in buffer for 7 days 21

S10.2 Additional TEM images of pre-aggregated $(GA)_{10}$ treated with buffer for 28 days 22

S10.3 Additional TEM images of pre-aggregated $(GA)_{10}$ treated with PEG-ME for 28 days 23

S10.4 Additional TEM images of pre-aggregated $(GA)_{10}$ treated with mPEG-L-CGGG-$(GA)_{10}$ for 28 days 24

S10.5 Additional TEM images of pre-aggregated $(GA)_{10}$ treated with mPEG-D-CGGG-$(GA)_{10}$ for 28 days 25

S1. $(GA)_{10}$ characterization

S1.1 $(GA)_{10}$ structure and purity

The structure of $(GA)_{10}$ was confirmed by ^1H NMR spectroscopy performed in DMSO-d6 and by MALDI-TOF mass spectrometry, while the purity was determined by HPLC. The expected peaks are present in the expected abundance in the NMR spectra, and the mass is as expected by MALDI. Although the HPLC chromatogram contains two peaks, the primary peak accounts for >91% of the total peak area. Due to the low solubility of the $(GA)_{10}$ peptide, we did not attempt to further purify by preparative-scale HPLC and instead used the peptide as synthesized.

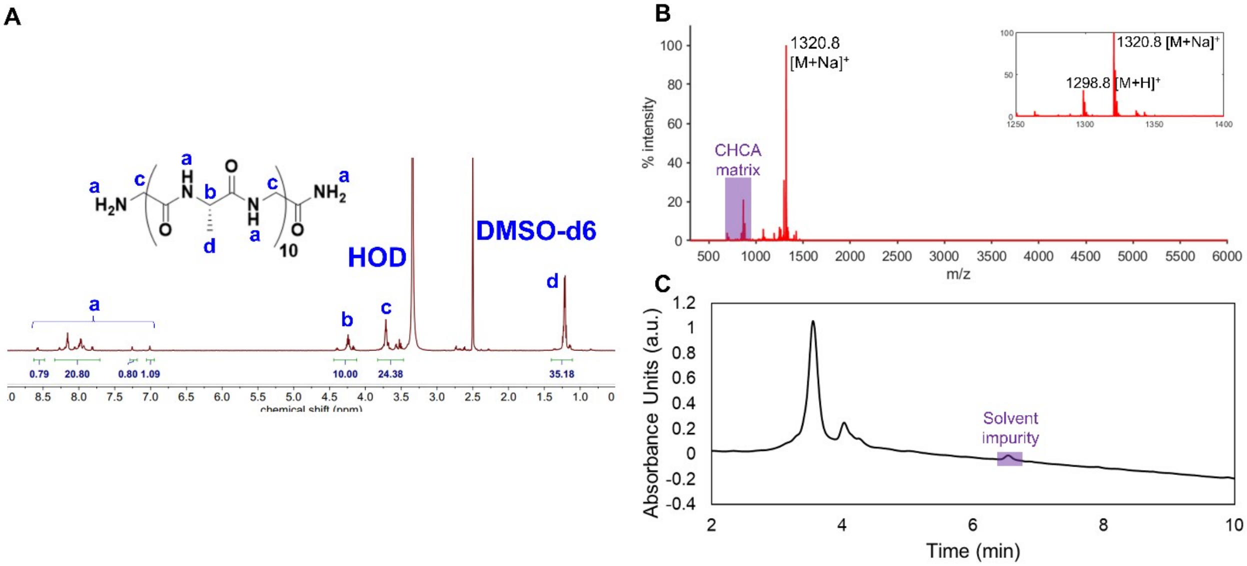

Figure S1. Characterization of $(GA)_{10}$: A) ^1H NMR (600 MHz, DMSO-d6) spectrum of $(GA)_{10}$; B) MALDI-TOF mass spectrum of $(GA)_{10}$ showing $[M+H]^+$ and $[M+Na]^+$ peaks; and C) Analytical HPLC chromatogram of $(GA)_{10}$. The primary HPLC peak accounts for >91% of the total peak area. The peptide was eluted on a linear AB gradient from 5% to 95% B (v/v) over 9 min, where A is ultrapure water + 0.1% TFA and B is acetonitrile + 0.1% TFA.

S1.2 $(GA)_{10}$ secondary structure

We used IR spectroscopy to determine the secondary structure of $(GA)_{10}$ in both the powder form (**Figure S2**) and in 10 mM phosphate buffer (**Figure 2**). In both forms, a peak at 1624 cm^{-1} characteristic of β-sheet formation is present, indicating that $(GA)_{10}$ is rich in β-sheet content.

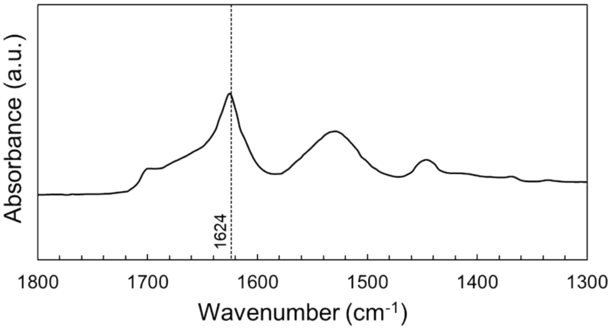

Figure S2. IR spectrum of powder $(GA)_{10}$ containing a peak at 1624 cm^{-1} characteristic of β-sheets.

S1.3 $(GA)_{10}$ forms turbid solutions at 3 mg/mL in 10 mM phosphate buffer

Upon addition of $(GA)_{10}$ to 10 mM phosphate buffer at 3 mg/mL, $(GA)_{10}$ immediately forms a turbid solution. The photos in **Figure S3** were taken within minutes of mixing $(GA)_{10}$ in the buffer.

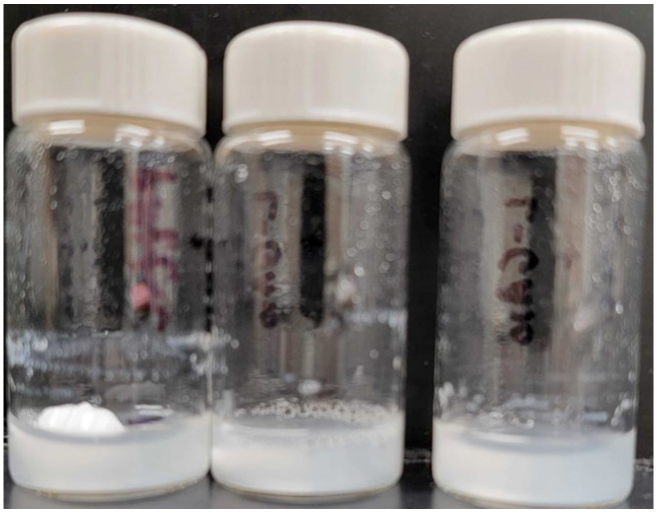

Figure S3. Photos of $(GA)_{10}$ at 3 mg/mL in 10 mM phosphate buffer taken minutes after initial mixing.

S2. TEM images of 10 mM phosphate buffer

Images taken of 10 mM phosphate buffer indicate no structure formation, so none of the features observed in any of the TEM images of samples can be attributed to the buffer (**Figure S4**).

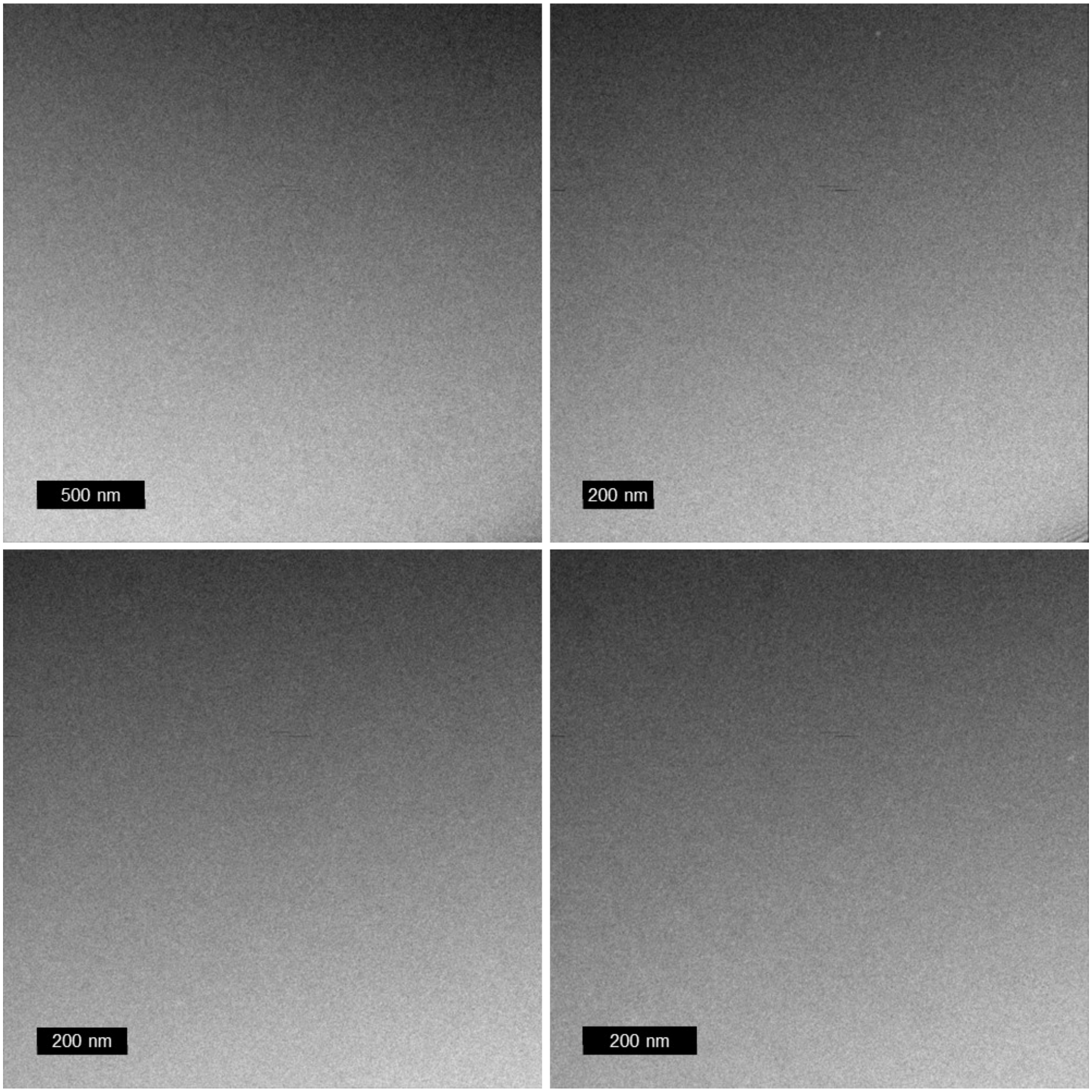

Figure S4. TEM images of 10 mM phosphate buffer show no signs of structure formation.

S3. Additional TEM images of $(GA)_{10}$

Figures S5 and **S6** show TEM images taken after overnight incubation of $(GA)_{10}$ in 10 mM phosphate buffer.

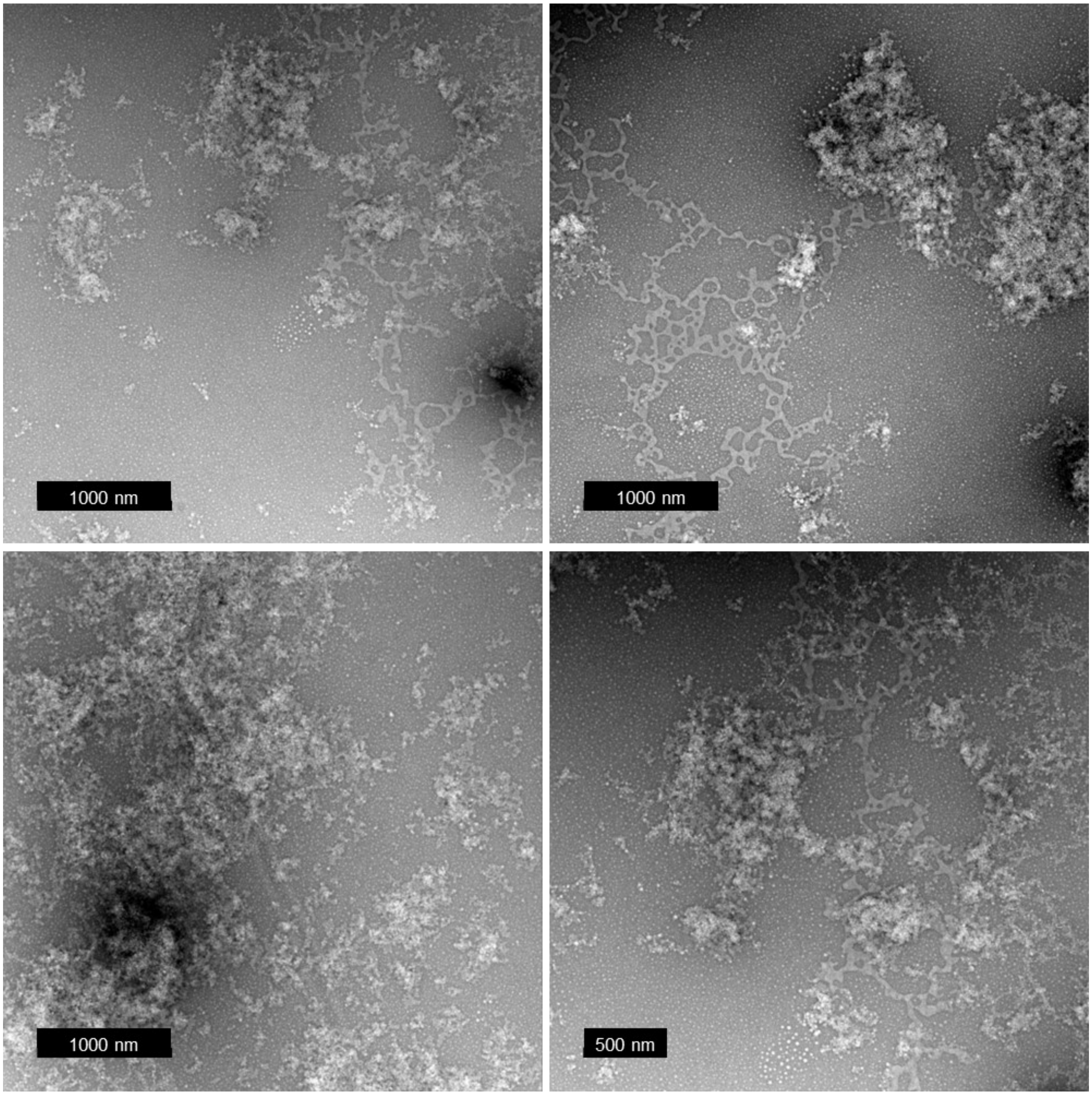

Figure S5. TEM images of $(GA)_{10}$ incubated in 10 mM phosphate buffer overnight. Aggregates of various sizes are observed, with smaller aggregates seemingly coming together to form larger aggregates.

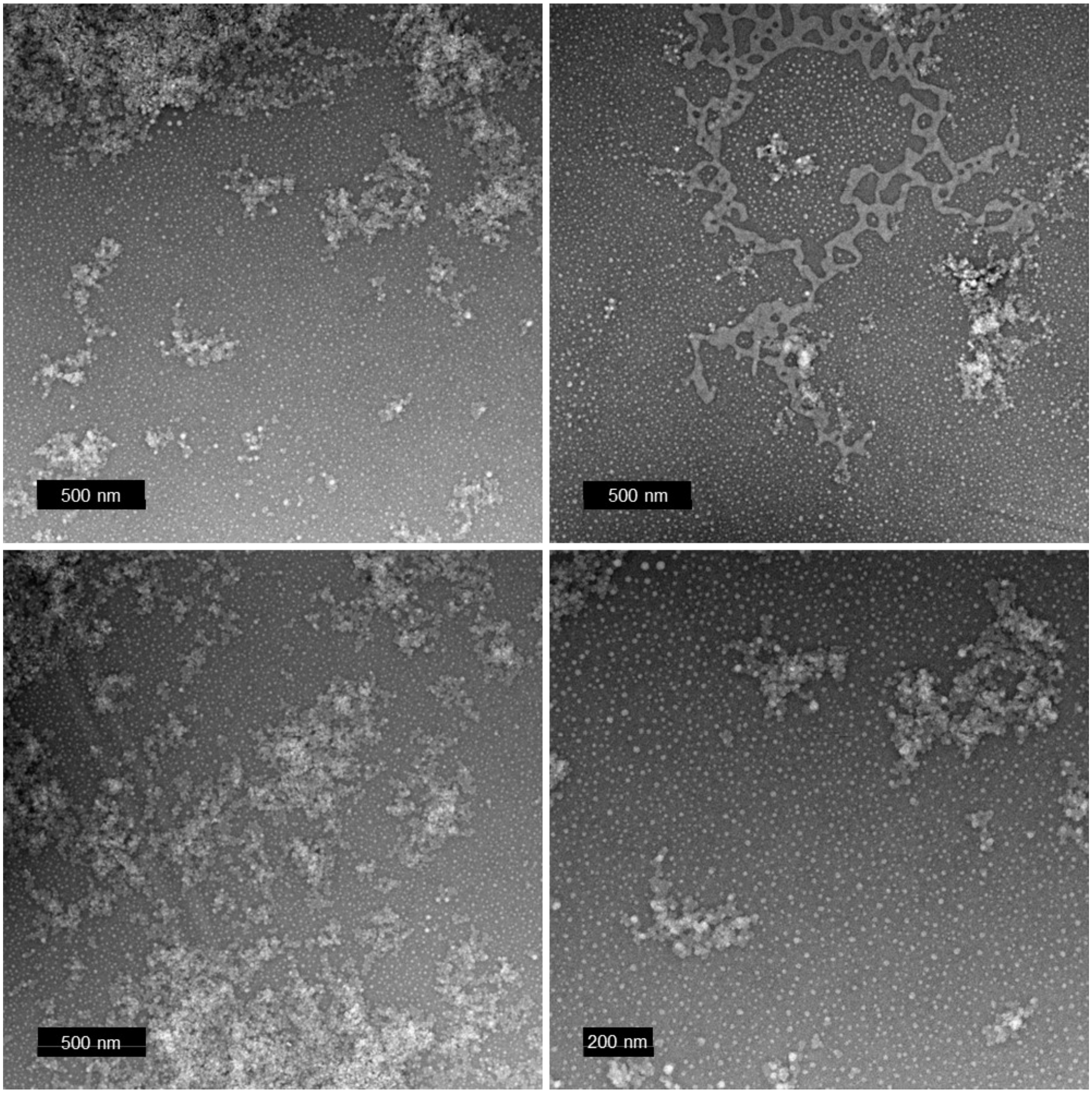

Figure S6. Additional TEM images of $(GA)_{10}$ incubated in 10 mM phosphate buffer overnight. Aggregates of various sizes are observed, with smaller aggregates seemingly coming together to form larger aggregates.

S4. Characterization of mPEG-CGGG-$(GA)_{10}$ conjugates

S4.1 Molar ratio required for full conversion of thiol-maleimide conjugation reaction

The thiol-maleimide conjugation reaction proceeds quickly under the reaction conditions but requires a high molar ratio of peptide: polymer. The HPLC traces of the reaction mixtures contain 4 peaks, corresponding to the peptide, polymer, newly formed conjugate, and another broad peak eluting after the polymer (**Figure S7**). The peptide is only sparingly soluble even under HPLC conditions, which we suspect contributes to a unique column interaction, causing the extremely

hydrophobic peptide that would be expected to elute late in the run to elute early instead. The fact that the peptide still has low solubility even under HPLC conditions indicates that they may remain assembled, and leads us to suspect that the broad peak that elutes after the polymer is the result of newly formed conjugate sequestering CGGG-$(GA)_{10}$ peptides in the reaction mixture before they react with polymer. Since some amount of peptide reactant is consumed by this interaction, more must be added to push the reaction to completion. Even at a molar ratio of 1.25:1 peptide: polymer, a large amount of unreacted polymer (mPEG-mal) remains. However, when pushed to 2:1 peptide: polymer, the polymer peak disappears almost completely, suggesting full reaction.

Two smaller peaks did not change with peptide:polymer ratio. The L-CGGG-$(GA)_{10}$ peak was consistently small, potential due to peptide that does not react with the polymer either interacting with the newly form conjugates or being filtered out prior to running HPLC. Additionally, there is a small peak that elutes just after the main polymer peak that does not change with molar ratio. This peak is also present in the chromatogram of the polymer alone, and thus is likely an impurity (the supplied mPEG-maleimide from Aldrich has a purity of >90%).

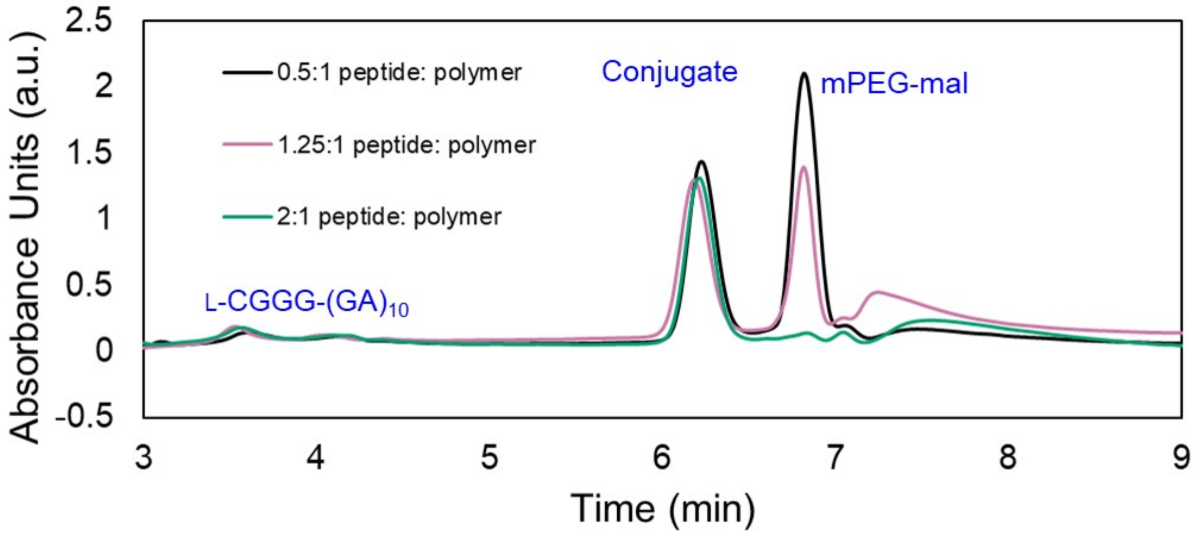

Figure S7. HPLC chromatograms of L-CGGG-$(GA)_{10}$ + mPEG-maleimide reaction mixtures at different peptide: polymer ratios showing peaks corresponding to the peptide, polymer, and newly formed conjugate. Only when reacted with a 2:1 excess of peptide: polymer is the polymer consumed completely. This may be due to the conjugate interacting with free peptide as it is formed, leading to the formation of the broad peak starting at ~7.3 min.

S4.2 Structure and purity of mPEG-CGGG-$(GA)_{10}$ conjugates

The conjugates purified by preparative-scale HPLC were analyzed by ^{1}H NMR spectroscopy to confirm the successful synthesis and SEC and HPLC to confirm purity. The expected peaks are present in the expected relative abundance (**Figure S8A**). By SEC, only one peak corresponding to the conjugate is present, indicating that preparative-scale HPLC successfully separated the conjugate from any unreacted peptide or polymer (**Figure S8B**). Finally, HPLC shows just one peak, indicating purity of the conjugates (**Figure S8C**).

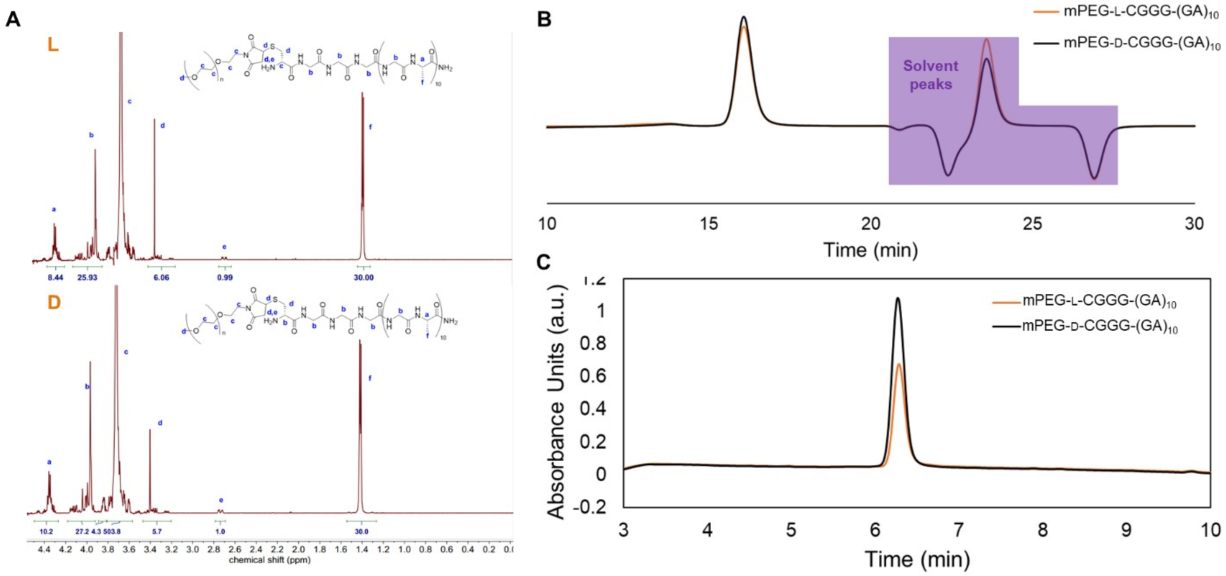

Figure S8. Characterization of mPEG-CGGG-(GA)$_{10}$ conjugates. A) ^{1}H NMR (600 MHz, D_2O) spectrum of D- and L-CGGG-(GA)$_{10}$. B) SEC and C) HPLC chromatograms showing D- and L-CGGG-(GA)$_{10}$ to elute as a single peaks with no discernible unreacted polymer or peptide impurities. With 1 proton on the polymer (i.e., signal e) set to 1, we can calculate n as the integration of polymer-associated c peaks (503.8 + 4.3 = 508) – 4 end group c protons = 504, divided by 4 protons per repeating unit = 126 repeating units. Thus, the number-average molecular weight of the conjugate = 126 (44.05 g/mol) + end group (peptide with CGGG linker - methoxy ethyl maleimide adduct = 1727.8 g/mol) = 7278 g/mol.

S4.3 Additional TEM images of mPEG-L-CGGG-$(GA)_{10}$

The additional TEM images in **Figure S9** were taken after overnight incubation in 10 mM phosphate buffer.

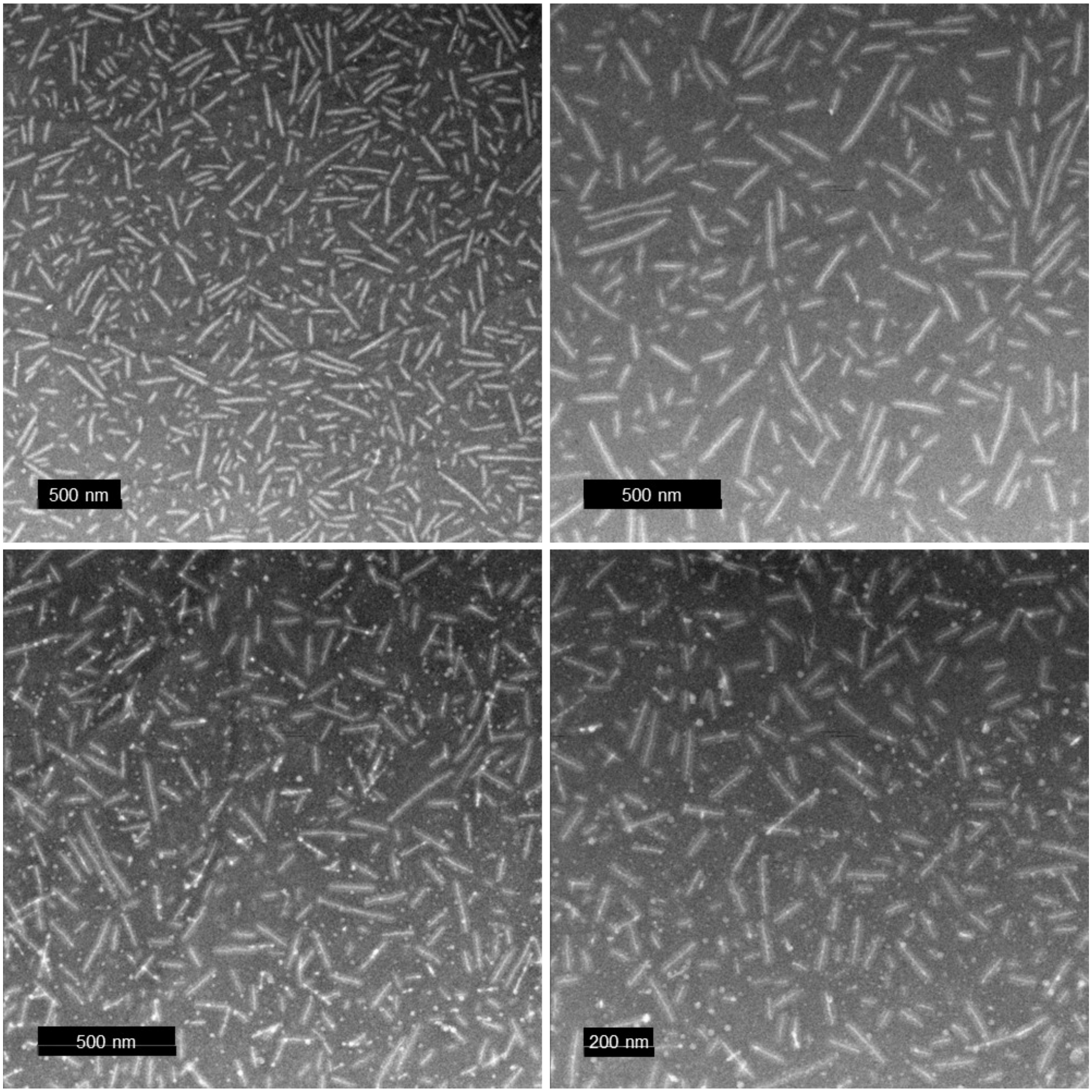

Figure S9. TEM images of mPEG-L-CGGG-$(GA)_{10}$ incubated overnight in 10 mM phosphate buffer. The most prevalent morphology is rod-like, leading to the proposed cylindrical micelle structure of the conjugates.

S4.4 Additional TEM images of mPEG-D-CGGG-$(GA)_{10}$

The TEM images in **Figure S10** were taken after overnight incubation in 10 mM phosphate buffer.

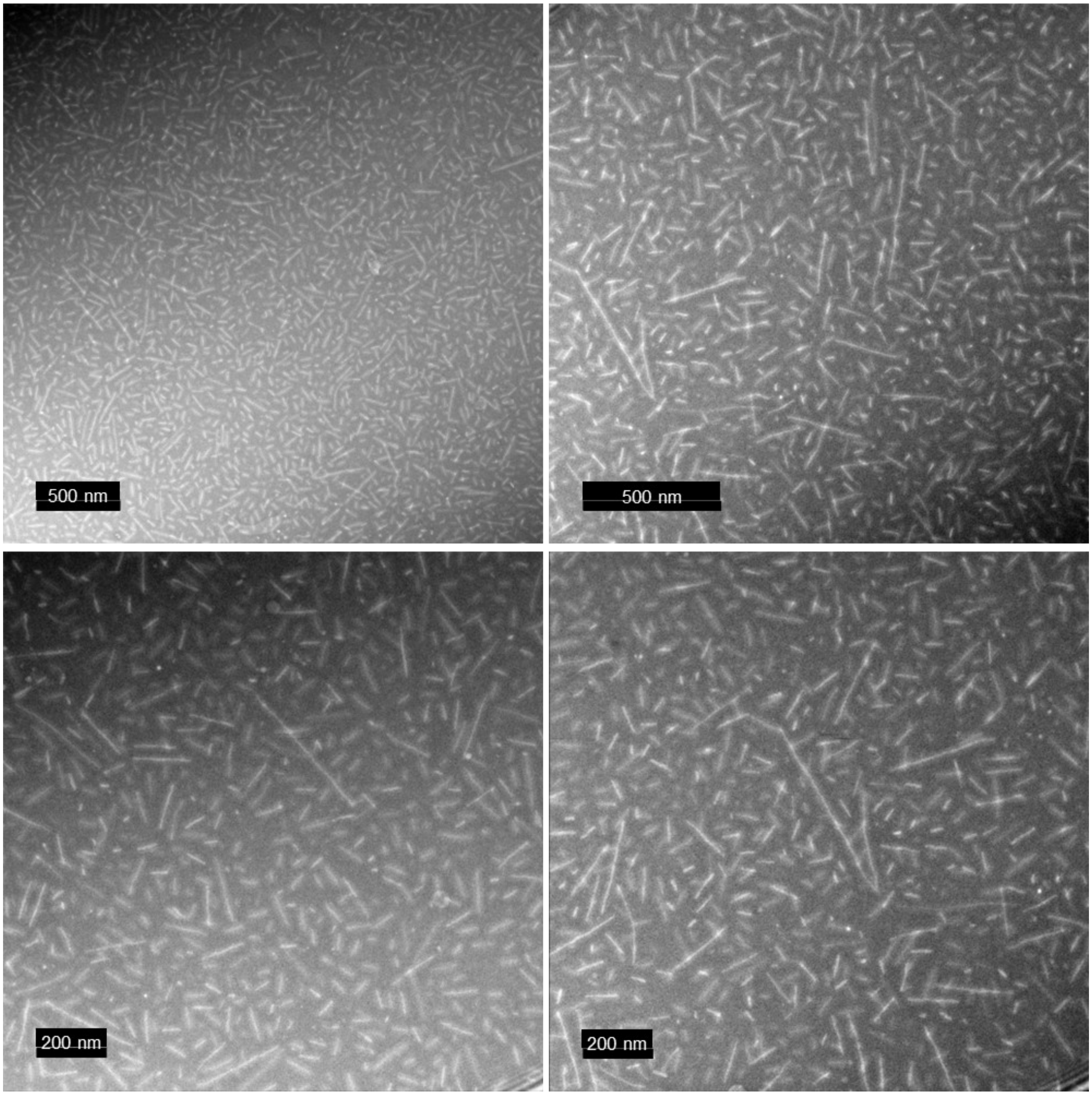

Figure S10. TEM images of mPEG-D-CGGG-$(GA)_{10}$ incubated overnight in 10 mM phosphate buffer. The most prevalent morphology is rod-like, leading to the proposed cylindrical micelle structure of the conjugates.

S5. Turbidity of mPEG-L-CGGG-$(GA)_{10}$ and mPEG-D-CGGG-$(GA)_{10}$ in 10 mM phosphate buffer

While the conjugates appear to fully dissolve by eye, we measured the turbidity of 7 mg/mL mPEG-L-CGGG-$(GA)_{10}$ and mPEG-D-CGGG-$(GA)_{10}$ minutes after dissolving in 10 mM phosphate buffer (**Figure S11**). The turbidity of the conjugate solutions was on the same order of magnitude

as the buffer alone, which is two or more orders of magnitude lower than the turbidities measured in the $(GA)_{10}$ sequestration experiments. Therefore, the turbidity of the conjugates alone does not significantly contribute to the data collected in any of the turbidity studies conducted.

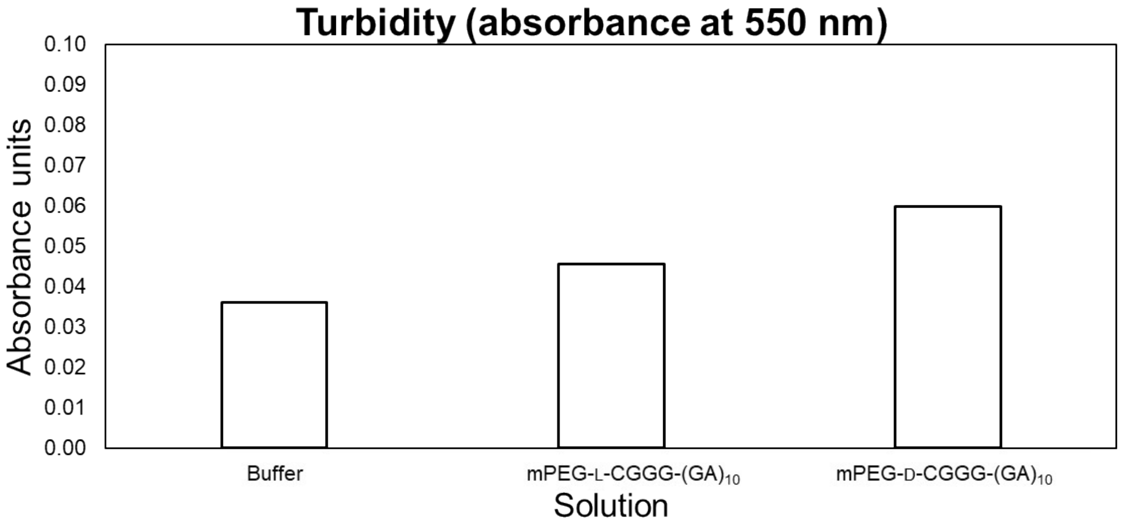

Figure S11. Turbidities of buffer, 7 mg/mL mPEG-L-CGGG-$(GA)_{10}$, and 7 mg/mL mPEG-D-CGGG-$(GA)_{10}$ in 10 mM phosphate buffer. These turbidities are multiple orders of magnitude lower than those in the turbidity experiments monitoring $(GA)_{10}$ sequestration.

S6. Turbidity of 10 mM phosphate buffer over 28 days

To test whether the turbidity of buffer solutions changes with time over the course of sequestration experiments (due to, for example, the growth of bacteria/microbes in aqueous solution), we monitored the turbidity of a 10 mM phosphate buffer solution over 28 days (**Figure S12**). We observed little change over the 28 days of the experiment, indicating that turbidity is not artificially increased by changes to the solution over time.

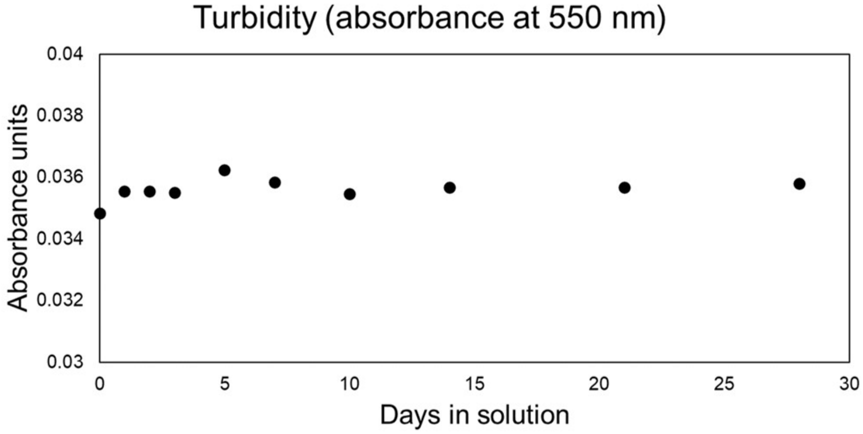

Figure S12. Turbidity of 10 mM phosphate buffer over 28 days. Little change is observed, indicating that changes in the buffer solution itself does not contribute to results observed in the turbidity experiments.

S7. Selection of a polymer control: Turbidity of $(GA)_{10}$ incubated with mPEG-mal and methyl ether-terminated PEG (PEG-ME)

Since we observed that $(GA)_{10}$ aggregation was limited in the presence of the maleimide-functionalized PEG (mPEG-mal) used in the conjugation reaction, we suspected the maleimide could be reacting with the $(GA)_{10}$ amines. Thus, we compared the turbidity of $(GA)_{10}$ incubated with mPEG-mal 5,000 to the turbidity of $(GA)_{10}$ incubated with PEG-ME 5,000, which does not have a reactive functional group (**Figure S13**). While incubating $(GA)_{10}$ with mPEG-mal yields initial increases in turbidity, but then relatively constant turbidity thereafter, $(GA)_{10}$ incubated with non-reactive PEG-ME results in continual increases in turbidity similar to $(GA)_{10}$ incubated in buffer alone. This comparison is consistent with the maleimide group reacting with and thereby preventing $(GA)_{10}$ aggregation. As our final conjugates do not have free maleimides, we opted for the non-reactive PEG-ME as the polymer control, as it better represents the conjugate without peptide present.

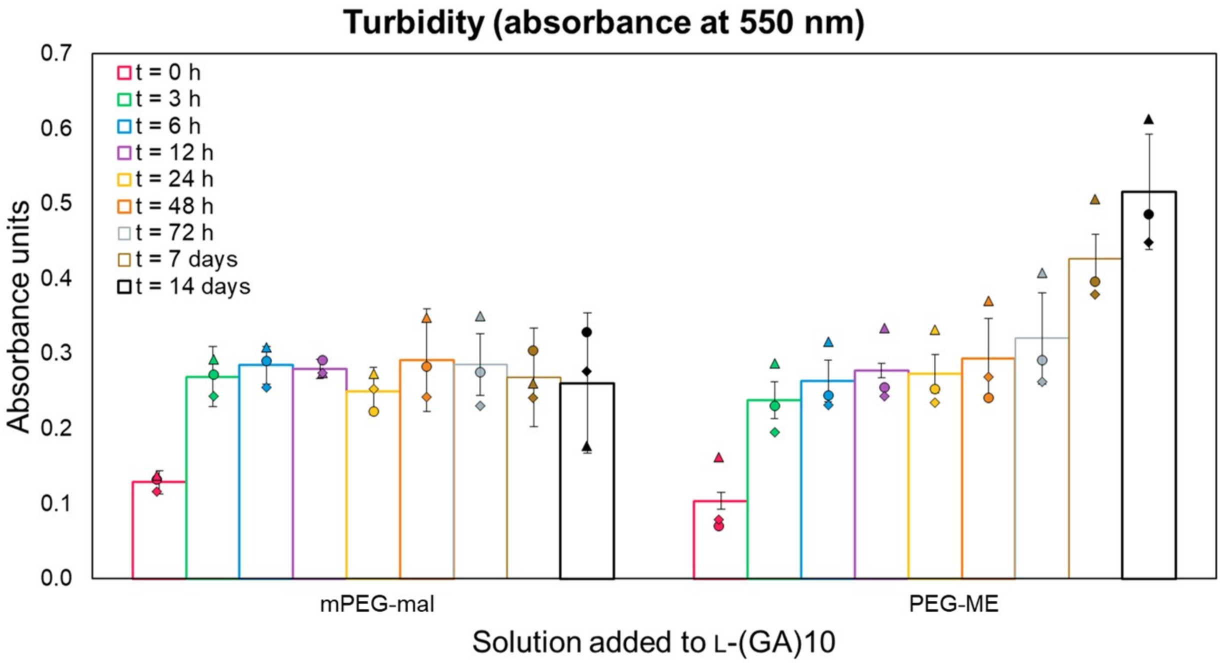

Figure S13. Turbidity of $(GA)_{10}$ incubated with mPEG-mal or PEG-ME. The $(GA)_{10}$ incubated with mPEG-mal initially increases in turbidity but then does not aggregate further, while the $(GA)_{10}$ incubated with PEG-ME continually increases in turbidity similar to its aggregation in buffer. This indicates that the unreacted maleimide contributes (likely by reaction) to preventing aggregation, not the PEG itself.

S8. Thioflavin T fluorescence of $(GA)_{10}$ incubated with buffer, mPEG-mal, mPEG-L-CGGG-$(GA)_{10}$, or mPEG-D-CGGG-$(GA)_{10}$

Concurrently with the turbidity experiments, we measured thioflavin T fluorescence for each vial (**Figure S14**), but after 2 days, saw little change with time. Taken together with the turbidity data and TEM indicating aggregation occurs, we suspect that the thioflavin T is serving mainly as an indicator of $(GA)_{10}$ accessible to the dye, whether in solution or in aggregates.

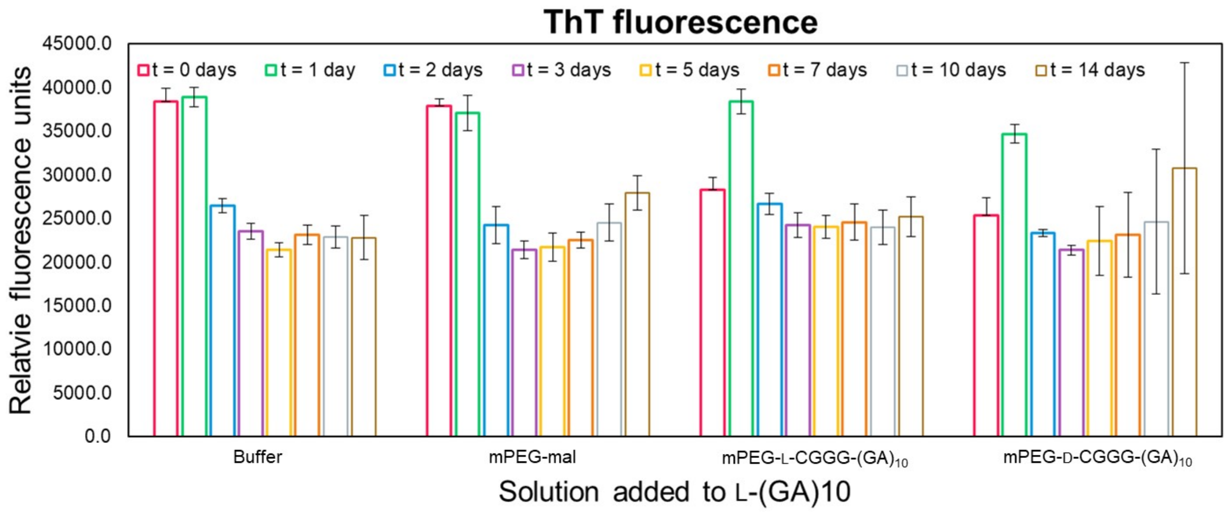

Figure S14. Thioflavin T fluorescence of $(GA)_{10}$ incubated with buffer, mPEG-mal, mPEG-L-CGGG-$(GA)_{10}$, or mPEG-D-CGGG-$(GA)_{10}$ in 10 mM phosphate buffer.

S9. Additional TEM images of coincubation study of $(GA)_{10}$ with buffer, mPEG-L-CGGG-$(GA)_{10}$, and mPEG-D-CGGG-$(GA)_{10}$

S9.1 Additional TEM images of $(GA)_{10}$ incubated in buffer at t = 0 days

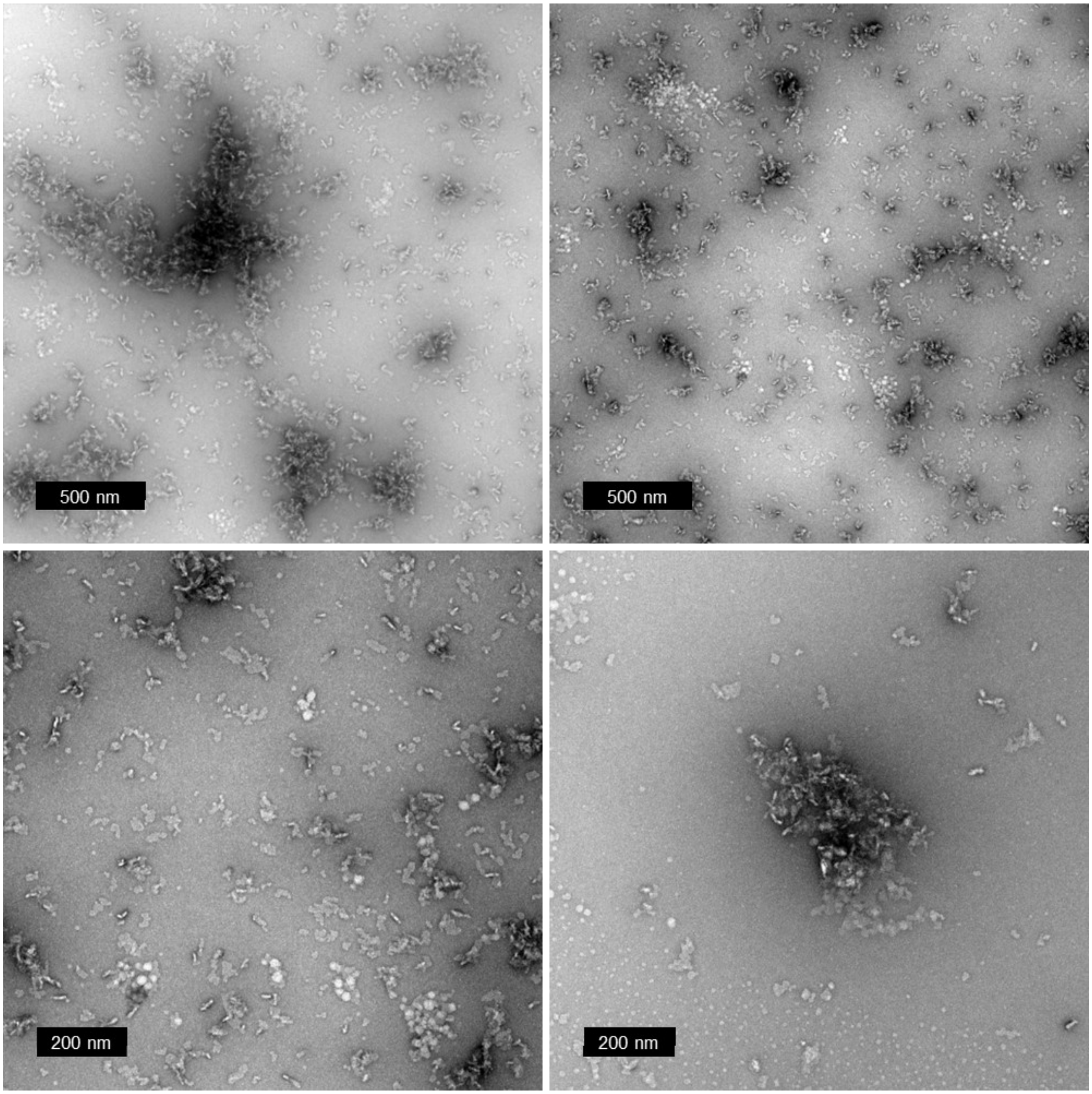

Figure S15. TEM images of $(GA)_{10}$ incubated in buffer for several minutes showing small aggregates, some of which come together into larger aggregates.

S9.2 Additional TEM images of $(GA)_{10}$ incubated in buffer at t = 14 days

After 14 days, larger $(GA)_{10}$ aggregates form (**Figure S17**). We noted several instances of irregularly shaped larger aggregates such as that in the center of the bottom right image. Further testing would be needed to confirm that such larger structures arise solely from $(GA)_{10}$ aggregation.

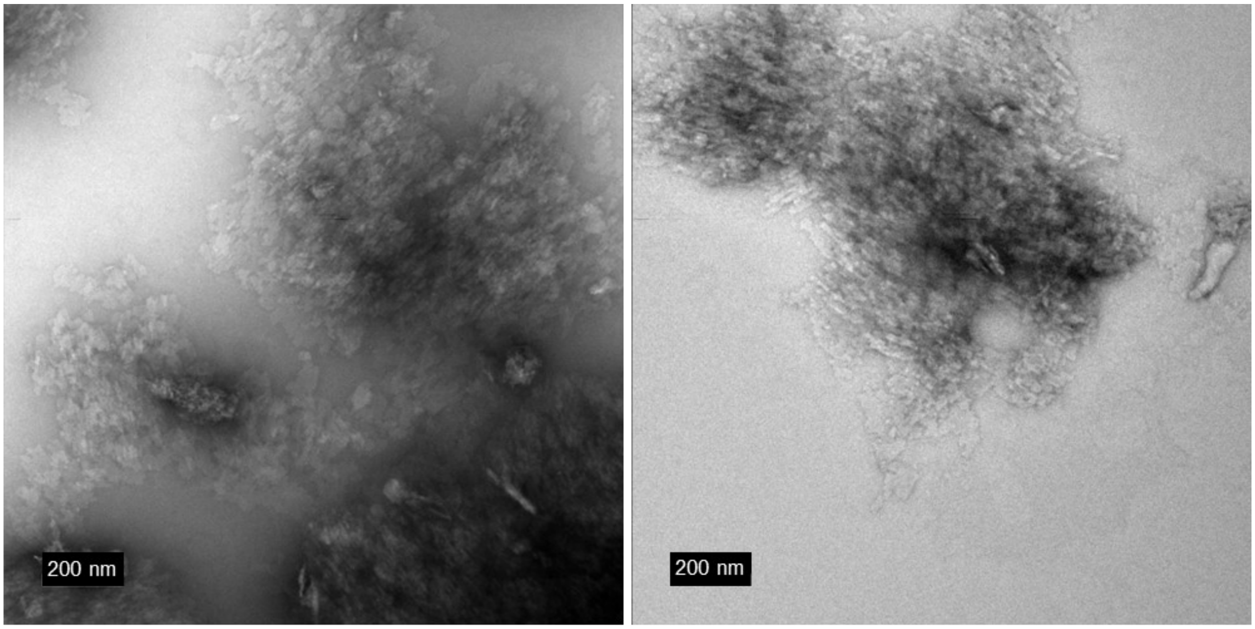

Figure S16. TEM images of $(GA)_{10}$ incubated in buffer for 14 days showing large aggregates.

S9.3 Additional TEM images of $(GA)_{10}$ incubated with mPEG-L-CGGG-$(GA)_{10}$ at t = 0 days

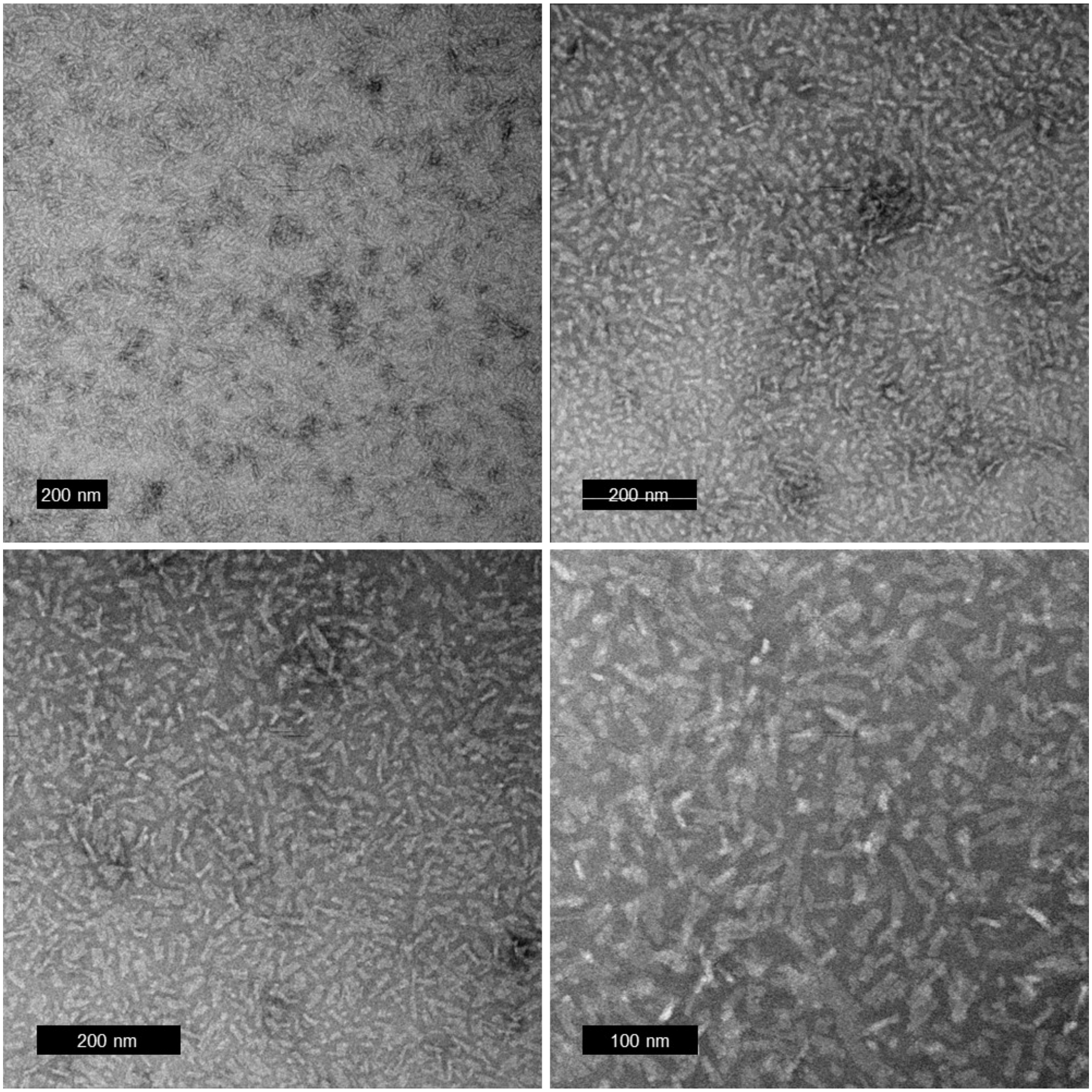

Figure S17. TEM images of $(GA)_{10}$ incubated in mPEG-L-CGGG-$(GA)_{10}$ for several minutes showing a mixture of small aggregates and rod-like conjugates.

S9.4 Additional TEM images of $(GA)_{10}$ incubated with mPEG-L-CGGG-$(GA)_{10}$ at t = 14 days

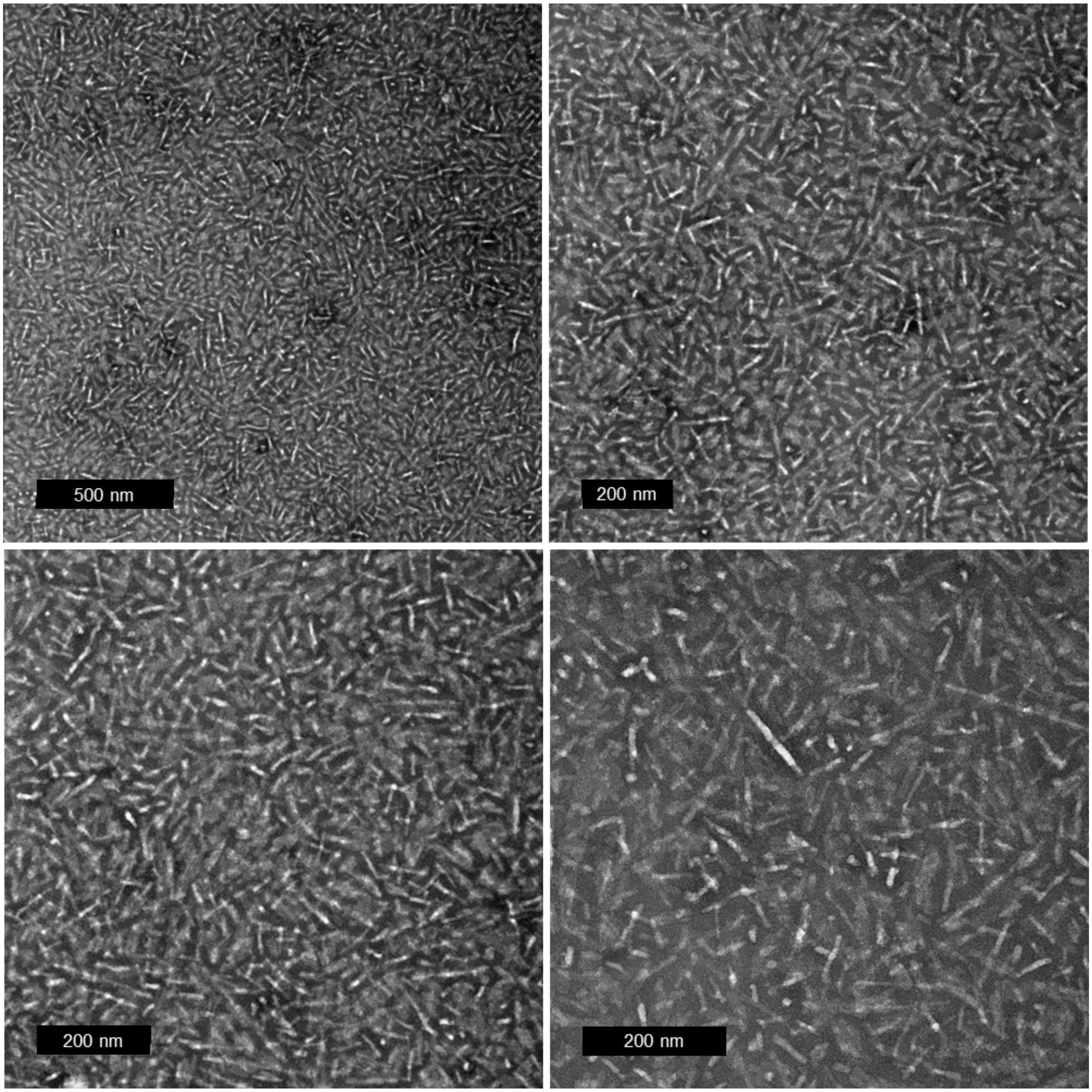

Figure S18. TEM images of $(GA)_{10}$ incubated in mPEG-L-CGGG-$(GA)_{10}$ for 14 days. These show a mixture of small aggregates and rod-like conjugates, but no large aggregates on the scale of what is seen when $(GA)_{10}$ is incubated in buffer alone.

S9.5 Additional TEM images of $(GA)_{10}$ incubated with mPEG-D-CGGG-$(GA)_{10}$ at t = 0 days

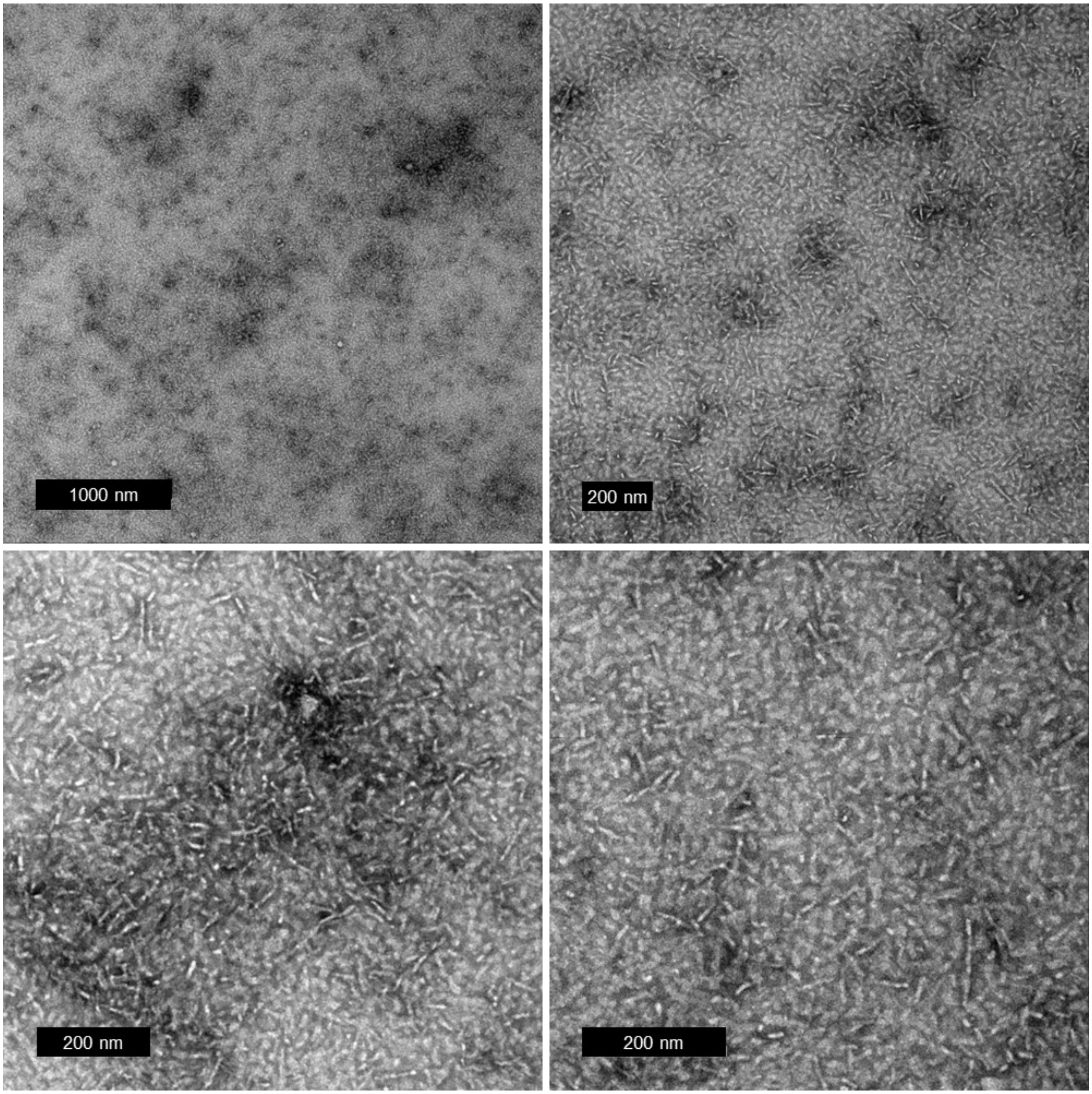

Figure S19. TEM images of $(GA)_{10}$ incubated in mPEG-D-CGGG-$(GA)_{10}$ for several minutes showing a mixture of small aggregates and rod-like conjugates.

S9.6 Additional TEM images of $(GA)_{10}$ incubated with mPEG-D-CGGG-$(GA)_{10}$ at t = 14 days

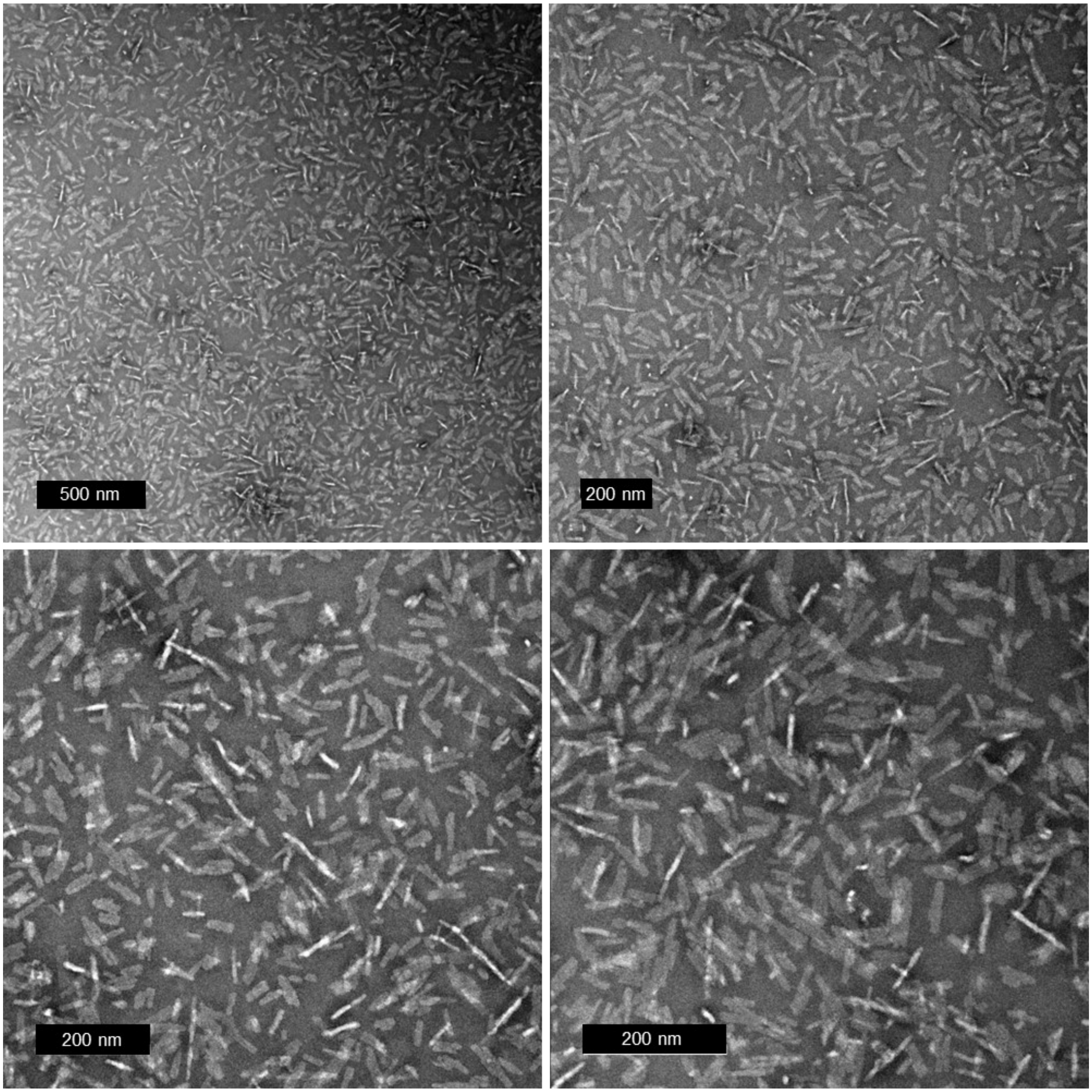

Figure S20. TEM images of $(GA)_{10}$ incubated in mPEG-D-CGGG-$(GA)_{10}$ for 14 days. These show a mixture of small aggregates and rod-like conjugates, but no large aggregates on the scale of what is seen when $(GA)_{10}$ is incubated in buffer alone.

S10. Additional TEM images of pre-aggregation study of $(GA)_{10}$ with buffer, PEG-ME, mPEG-L-CGGG-$(GA)_{10}$, and mPEG-D-CGGG-$(GA)_{10}$

S10.1 Additional TEM images of $(GA)_{10}$ pre-aggregated in buffer for 7 days

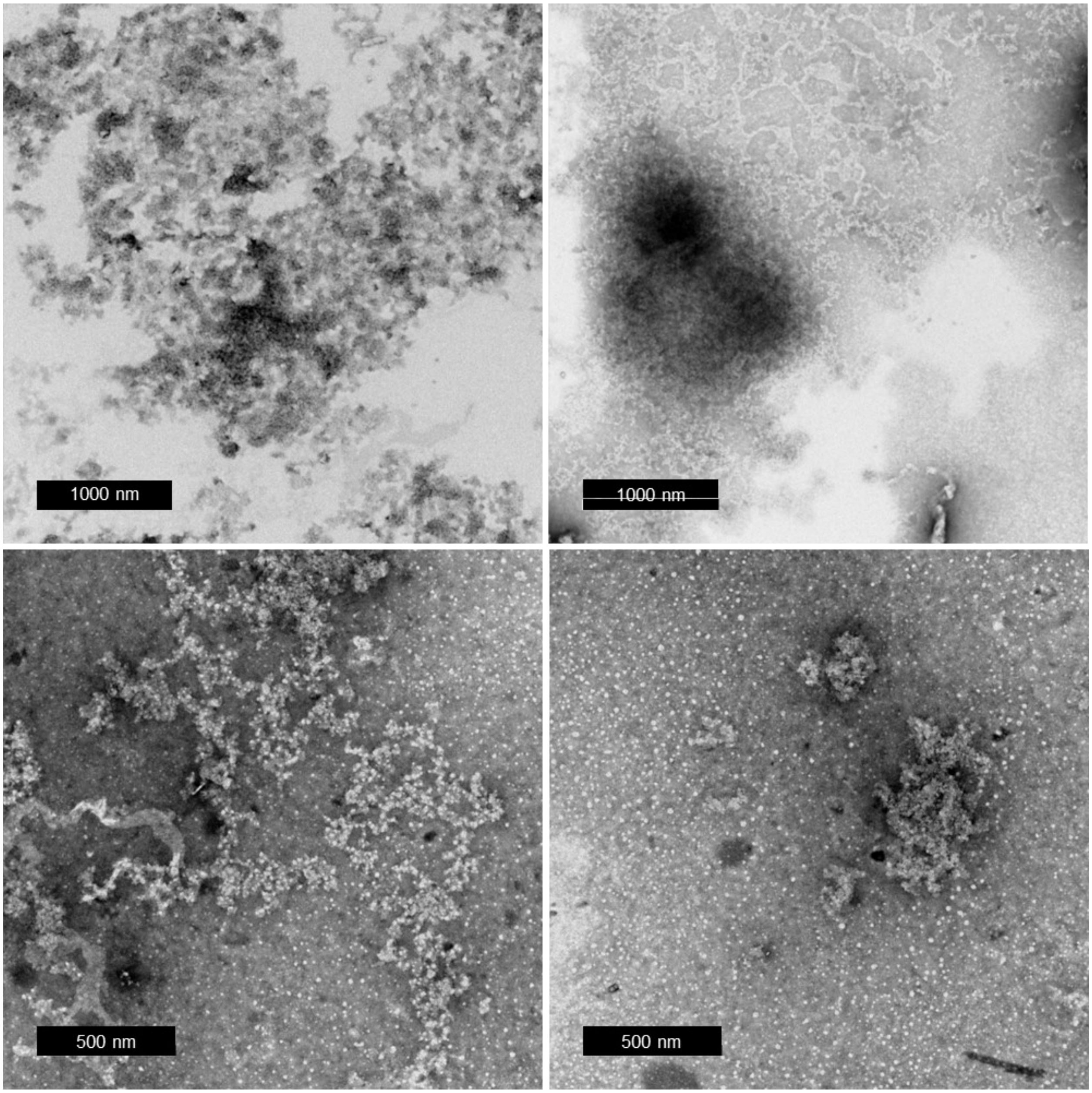

Figure S21. TEM images of $(GA)_{10}$ after 7 days of incubation in 10 mM phosphate buffer. These images show a combination of small, medium, and large aggregates, with sizes in between $(GA)_{10}$ structures incubated in buffer for a few minutes and 14 days.

S10.2 Additional TEM images of pre-aggregated $(GA)_{10}$ treated with buffer for 28 days

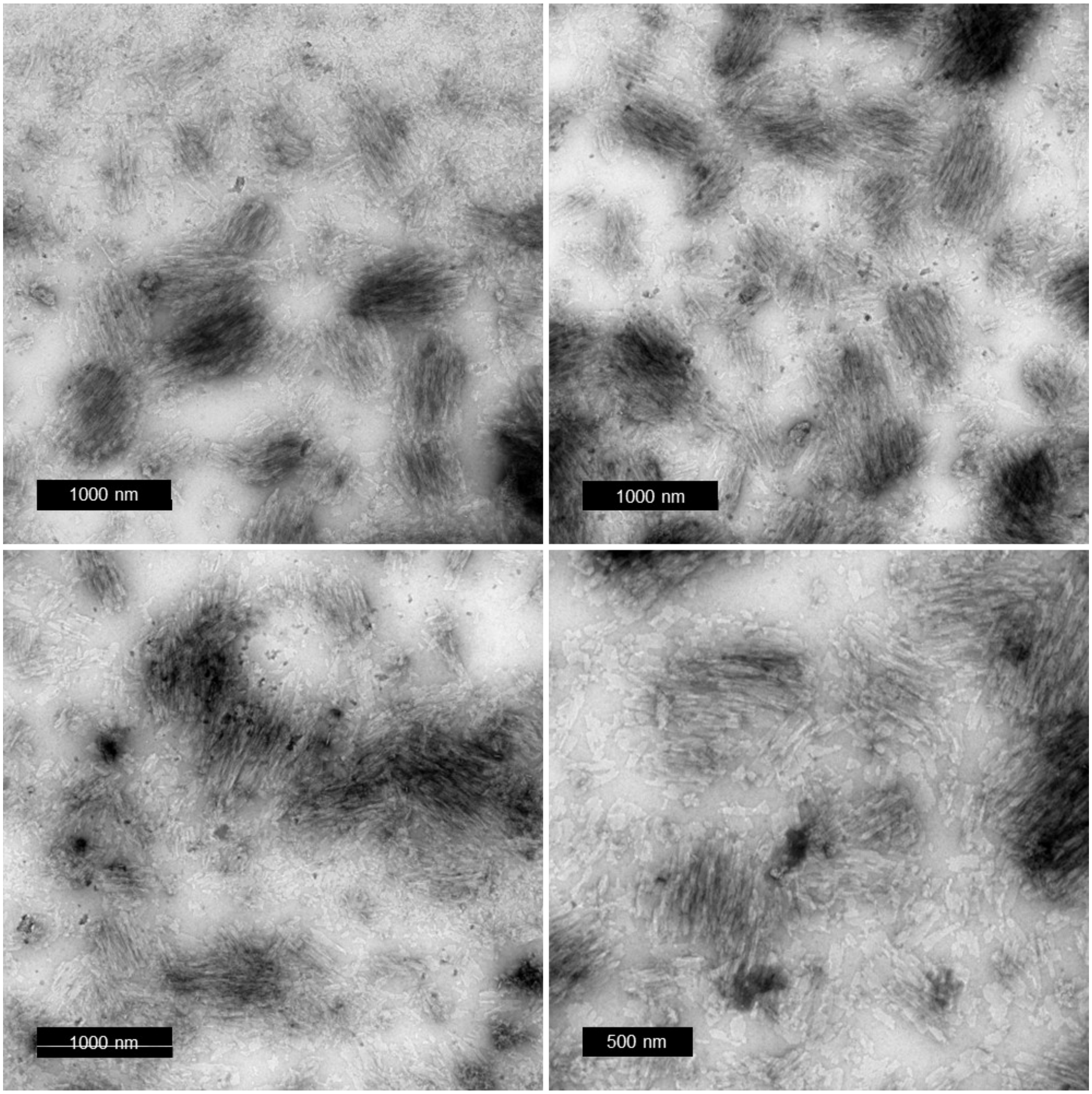

Figure S22. TEM images of pre-aggregated $(GA)_{10}$ incubated with buffer for 28 days, showing large aggregates.

S10.3 Additional TEM images of pre-aggregated $(GA)_{10}$ treated with PEG-ME for 28 days

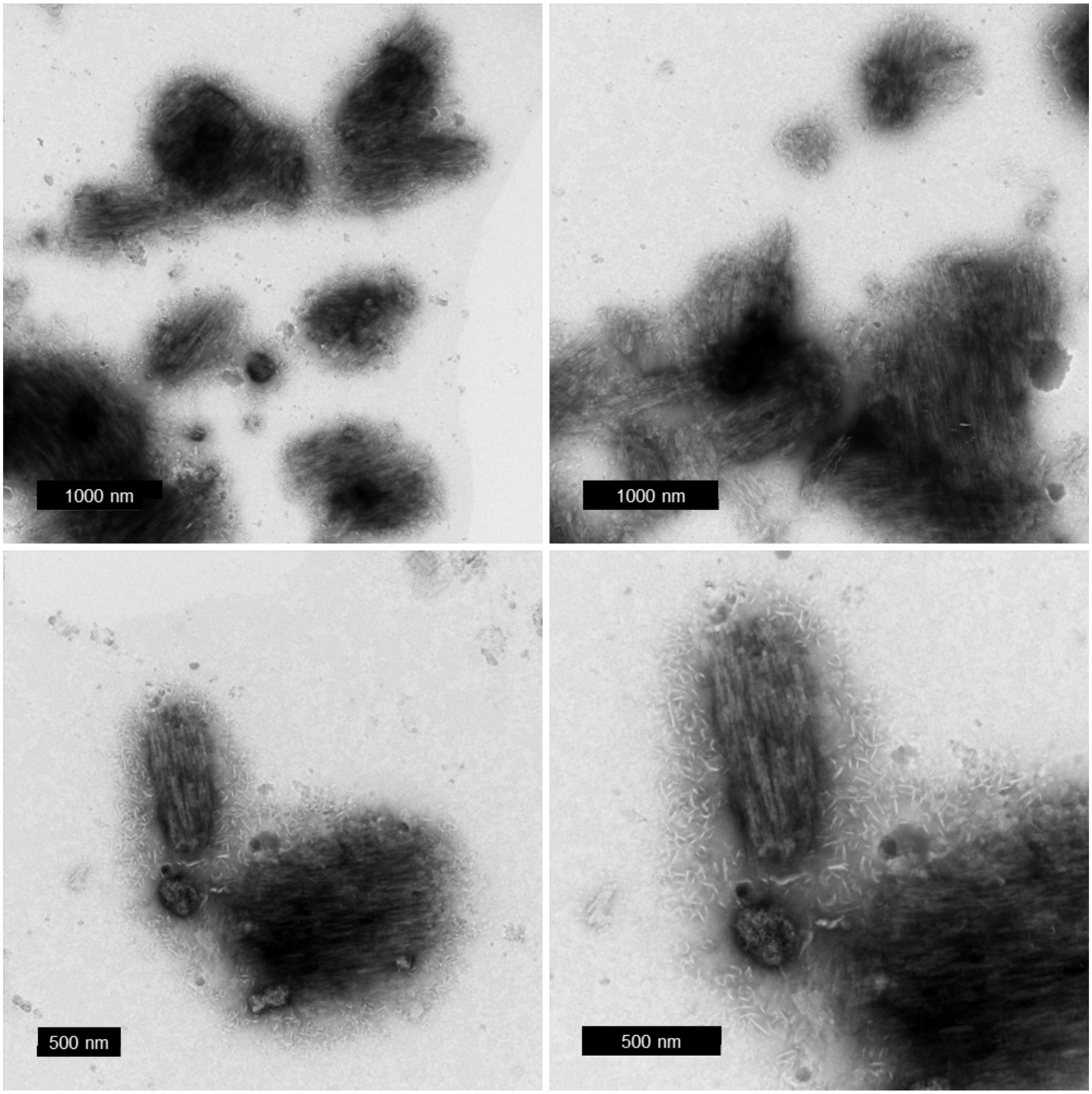

Figure S23. TEM images of pre-aggregated $(GA)_{10}$ incubated with PEG-ME for 28 days, showing large aggregates, similar in size to the aggregates observed for the pre-aggregated $(GA)_{10}$ treated with buffer.

S10.4 Additional TEM images of pre-aggregated $(GA)_{10}$ treated with mPEG-L-CGGG-$(GA)_{10}$ for 28 days

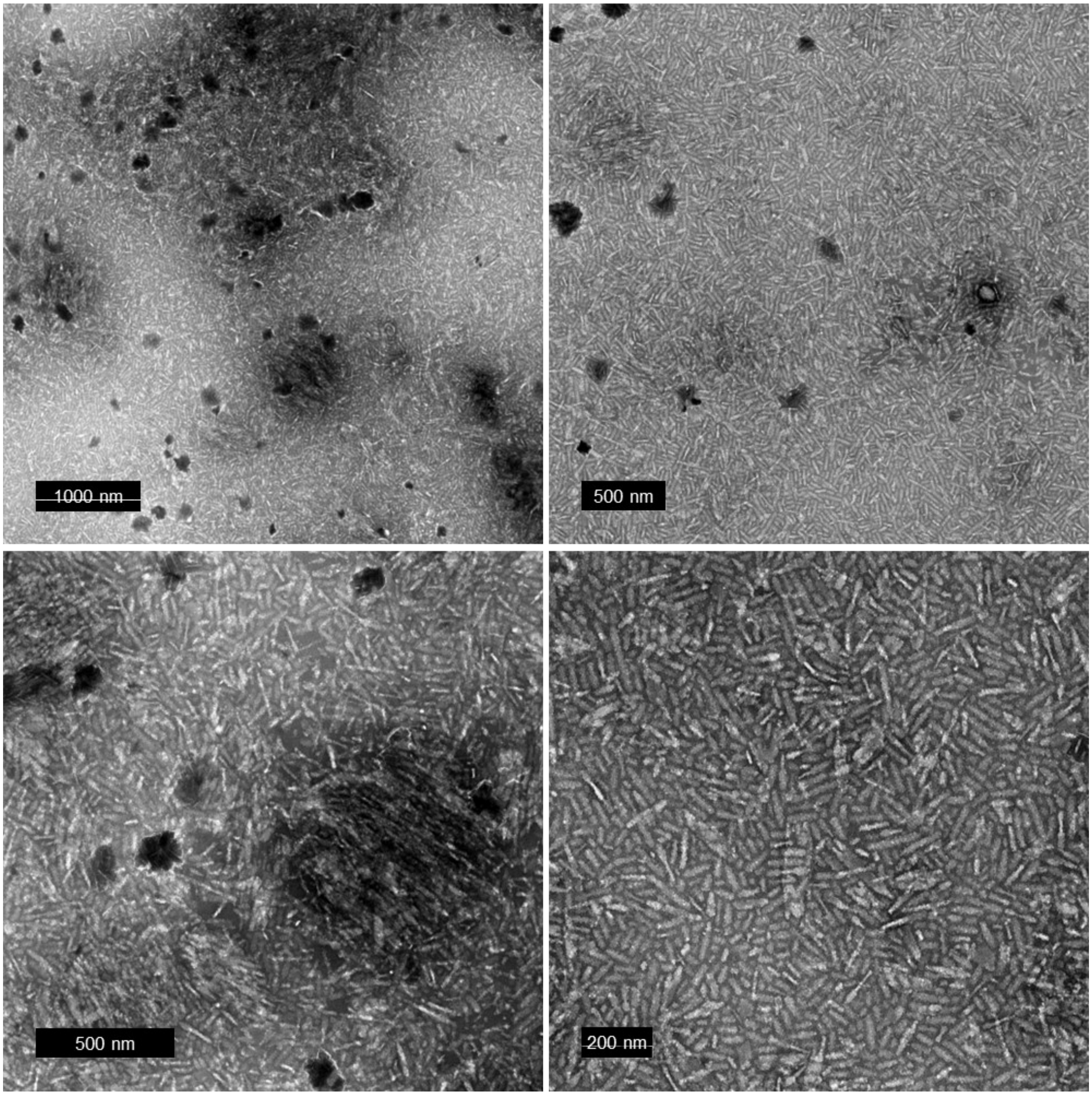

Figure S24. TEM images of pre-aggregated $(GA)_{10}$ incubated with mPEG-L-CGGG-$(GA)_{10}$ for 28 days. While the morphology is primarily rod-like micelles corresponding to the conjugate and some small aggregates, there are still areas with some larger aggregates.

S10.5 Additional TEM images of pre-aggregated $(GA)_{10}$ treated with mPEG-D-CGGG-$(GA)_{10}$ for 28 days

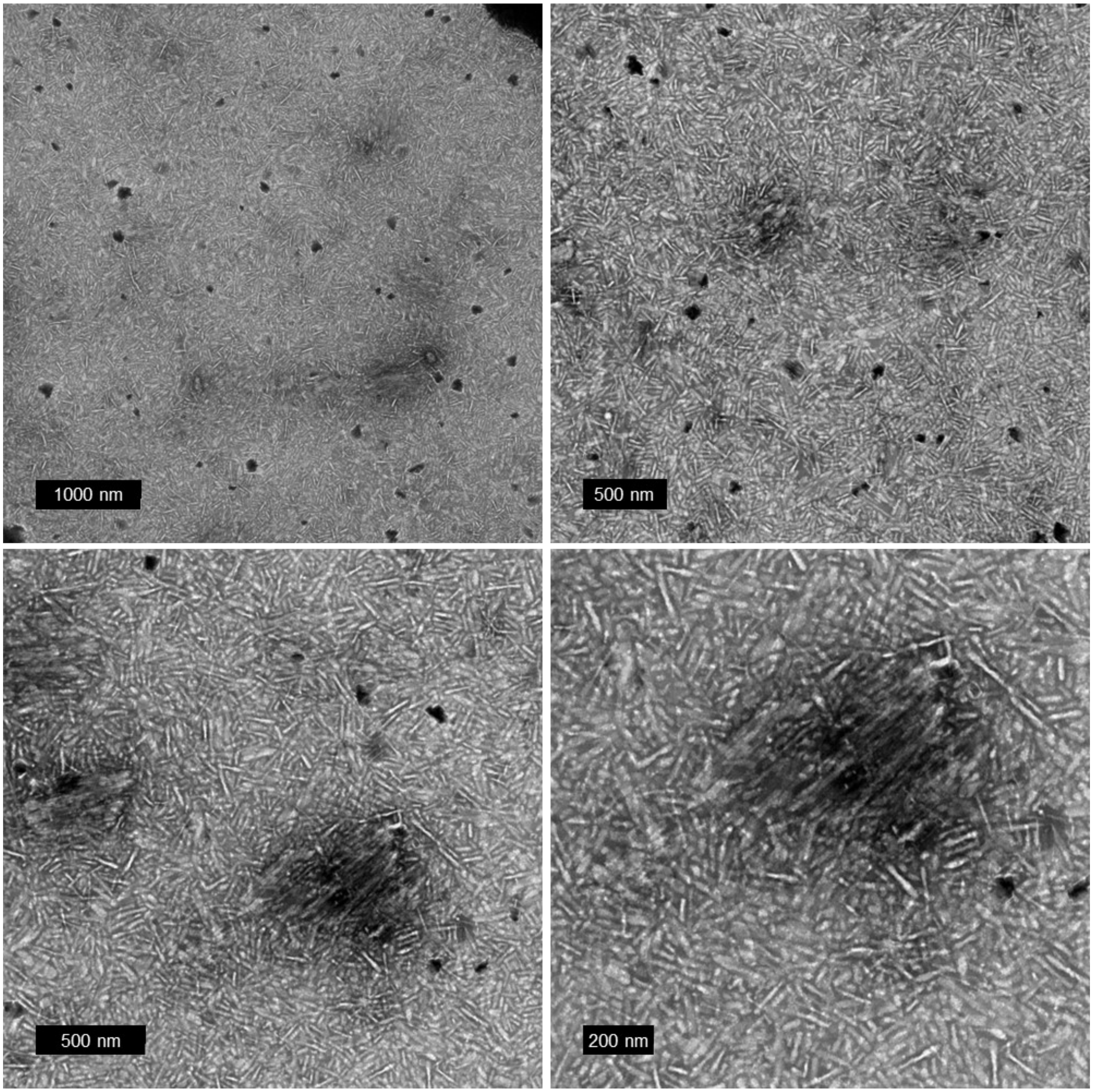

Figure S25. TEM images of pre-aggregated $(GA)_{10}$ incubated with mPEG-D-CGGG-$(GA)_{10}$ for 28 days. While the morphology is primarily rod-like micelles corresponding to the conjugate and some small aggregates, there are still areas with some larger aggregates.
